# Biological Evaluation of Newly Synthesized Biaryl Guanidine Derivatives to Arrest *β*-Secretase Enzymatic Activity Involved in Alzheimer's Disease

**DOI:** 10.1155/2020/8934289

**Published:** 2020-05-11

**Authors:** Sayyad Ali, Muhammad Hassham Hassan Bin Asad, Fahad Khan, Ghulam Murtaza, Albert A. Rizvanov, Jamshed Iqbal, Borhan Babak, Izhar Hussain

**Affiliations:** ^1^Department of Pharmacy, COMSATS University Islamabad, Abbottabad Campus, 22060, Pakistan; ^2^Department of Chemistry, Michigan State University, East Lansing, Michigan 48824, USA; ^3^Department of Genetics, Institute of Fundamental Medicine and Biology, Kazan Federal University, 420021, Russia; ^4^School of Packaging, Michigan State University, East Lansing, Michigan 48824-1223, USA; ^5^Department of Pharmacy, COMSATS University Islamabad, Lahore Campus, Pakistan

## Abstract

Proteases BACE1 (*β*-secretases) enzymes have been recognized as a promising target associated with Alzheimer's disease (AD). This study was carried out on the principles of molecular docking, chemical synthesis, and enzymatic inhibition of BACE1 enzymes via biaryl guanidine-based ligands. Based on virtual screening, thirteen different compounds were synthesized and subsequently evaluated via *in vitro* and *in vivo* studies. Among them, 1,3-bis(5,6-difluoropyridin-3-yl)guanidine (compound (9)) was found the most potent (IC_50_ = 97 ± 0.91 nM) and active to arrest (99%) *β*-secretase enzymes (FRET assay). Furthermore, it was found to improve the novel object recognition test and Morris water maze test significantly (*p* < 0.05). Improved pharmacokinetic parameters, viz., Log *P*_o/w_ (1.76), Log *S* (-2.73), and better penetration to the brain (BBB permeation) with zero Lipinski violation, made it possible to hit the BACE1 as a potential therapeutic source for AD.

## 1. Introduction

Alzheimer's disease (AD) is a continuous neurodegenerative disorder that leads to mental deterioration particularly in geriatric population. It is characterized by serious loss of cognition and social and psychiatric anomalies [1, 2]. Amyloid-*β* (A*β*) peptide deposition and agglutination of tau proteins are the main pathologic features of the disease that led to the inflammation and eventually loss of neurons [1, 2]. Indeed, A*β* peptide accumulation resulted from degradation of *β*-amyloid precursor proteins (APP) via *β* and *γ* secretase enzymes. The beta site APP cleaving enzyme (BACE1) (composed of 501 extracellular and 22 cytoplasmic amino acids domains) is a main player of producing A*β* plaques and a promising inhibiting target to control AD. The biological inhibition of BACE1 was focused to inhibit A*β* formation [2–4].

Statin-type structures were developed initially to lock the two aspartic acids in the catalytic domain; however, these ligands showed very low penetration into the brain. Later on, cyclic and rigid conformational structures (heterocyclic nucleus for enzyme inhibition) were recognized to improve blood-brain barrier (BBB) circulation [5, 6]. To the best of our knowledge, guanidine derivatives have been documented previously to antagonize nervous disorders, cited by Gerritz et al. as acyl guanidines [6, 7]. We substituted the “acyl” portion with biaryl moieties to find out its inhibition results on BACE1. The study was designed to synthesize various symmetrical biaryl guanidine derivatives with particular interest to incorporate fluorines or -CF_3_ moieties along with heterocyclic rings to the parent nucleus. The *in silico* study allows a noncovalent bonding interaction of the proposed fluorine and amide (–NH) group within the active site of BACE1 (Asp228 and Asp32); it extends to the Thr231, Tyr71, and Tyr 198 sites. Consequently, we proposed to insert substitutes that could cover the interaction in the S3 pocket. These results gave us a small library of ligands linked to numerous electron-donating and electron-withdrawing groups. These were chemically synthesized to analyze the outcome for BACE1 inhibition [8].

## 2. Material and Methods

### 2.1. *In Silico* Study

The *in silico* study was performed by utilizing the AutoDock Vina program. The X-ray crystallographic structure of BACE1 enzymes (PDB ID: 1FKN, 1.9 Å) was adopted from the Protein Data Bank (http://www.rcsb.org) [5, 9]. The addition of water, charges, and hydrogen atoms along with the removal of cocrystallized ligand-protein structures was prepared for molecular docking. The active site was specified via grids around the cocrystallized ligands before the removal of ligands. Gasteiger charges and polar hydrogen were contributed while steady charges were produced using MGL Tools (v 1.5.4). Proposed compounds were sketched and optimized with the ChemDraw Ultra (v 16.0.1, CambridgeSoft Corporation, USA) and MM2 ChemBioDraw software. A grid box (1.0 Å) was selected to cover up the catalytic site of an enzyme while coordinate centers were marked as 18.439, 4.370, and 9.75. The rest of all necessary parameters were recorded via an online server http://www.swissadme.ch [10].

BACE1 allosteric domains, grid box size (1.0 Å), and coordinates were specified as 50, 48, and 52. Moreover, nine poses (generated via the Lamarckian Genetic Algorithm based on binding free energies) were fixed and visualized with the PyMol molecular viewer (v 2.1, Schrödinger Inc., New York, NY, USA) and Discovery Studio (v 17.2, BIOVIA Corp., San Diego, CA, USA) software [11]. Ligand-enzyme interaction and various pharmacokinetic properties, i.e., Clog*P*, Lipinski, and BBB permeation, were obtained via an online server http://www.swissadme.ch [10]. All the proposed ligands were synthesized after a detailed *in silico* authentication.

### 2.2. Chemical Synthesis (N,N′-Biaryl Guanidines)

N,N′-Biaryl guanidine derivatives were synthesized via arylation of guanidine moieties according to the methods described by Hui et al. [12].

Briefly, Schlenk line flask mixture was composed of guanidine nitrate (1.0 mmol), aryl halides (1.0 mmol, Ar-I/Br), ligand N-methylglycine (8.9 mg/0.2 mmol for Ar-I; 17.8 mg/0.1 mmol for Ar-Br), recrystallized CuI (9.5 mg/0.05 mmol for Ar-I; 19 mg/0.1 mmol for Ar-Br), and K_3_PO_4_ (1.270 g/6 mmol) under described reaction conditions. Reaction mixture was evacuated and backfilled, and acetonitrile (5 ml) was added subsequently. It was continuously stirred until aryl halide was completely utilized (monitored periodically with TLC) and collected through ethyl acetate. Brine solution was added after separation of the upper organic layer and dried with Na_2_SO_4_. Furthermore, the material was purified through silica gel column chromatography with eluents methanol/methylene chloride in the ratio of 25 : 1-40 : 1 to acquire the final product(s).

### 2.3. Spectral Analysis and Other Parameters

The ^1^H-NMR, ^13^C-NMR, and ^19^F-NMR spectra were recorded by Agilent (DDR2 500 MHz NMR spectrometers) equipped with 7600AS 96 sample autosamplers running VnmrJ 3.2A. TMS was used as the internal standard while the chemical shift and coupling constant (*J*) were described in ppm (*δ*) and MHz, respectively. Other related information of the synthesized ligands (Clog*P*, Lipinski, BBB permeation, and pharmacokinetics) was obtained from an online server http://www.swissadme.ch/.

### 2.4. Fluorescence Resonance Energy Transfer (FRET) Assay

BACE1 inhibitory activity was carried out by adopting the procedures described by the Sigma-Aldrich FRET-based assay activity kit (product # CS0010).

Briefly, the assay was carried out in a fixed volume of 100 *μ*l with the BACE1 substrate (20 *μ*l, 50 *μ*M), assay buffer (78 *μ*l/78-*X* *μ*l, pH 4.5), test sample(s) (*X* *μ*l, 100 *μ*M), and BACE1 enzyme (2 *μ*l, ~0.3 unit/*μ*l) incubated at 37°C for 75 min in 96-well microplate reaction mixture [13]. The baseline fluorescence (null time) signal was noticed (at 320 nm) immediately after the addition of BACE1 enzymes while emission signals were monitored at 405 nm at 25°C. Both the enzyme and the substrate were prepared in the buffer while tested samples dissolved primarily in DMSO (5%) subsequently in serially diluted buffer solution in a desired volume of 2 *μ*l, 3 *μ*l, and 5 *μ*l having 200 pmol, 300 pmol, and 500 pmol concentration, respectively. A standard curve was prepared between fluorescent unit (FU) and standard solution (100 *μ*M, 100-500 pmol, and 1-5 *μ*l) to find out 50% BACE1 cleaving activity [14]. The sample blank (buffer with substrate) was treated as the negative control while the positive control was the solution of the buffer with substrate and enzyme mixture (http://www.sigmaaldrich.com). Finally, IC_50_ of the intended compounds were calculated via GraphPad Prism v8 software.

### 2.5. Experimental Animals

Male BALB/c mice (3.5 to 5.5 months of age) were purchased from National Institute of Health (NIH) Islamabad, Pakistan. All experiments were performed by following the rules and regulations in accordance with the Helsinki Declaration after getting permission from the Departmental Review Committee (Ref. # PHM.Eth/CS-M01/18-001 dated 05/2018). Animals were kept in standard cages and provided food, water, and cleanliness in accordance with the documented protocols. Moreover, they were categorized into nine groups (*n* = 10) for experimental purposes.

### 2.6. Animal Preparation

The control group of mice was treated with drinking water having aluminum chloride (17 mg/kg) and tape water for a period of five weeks. Synthesized ligands (20-55 mg/kg) were administered to neutralize the AlCl_3_ effects and to design treated mouse groups. All the behavioral tests were performed on days 28 to 34 (08:30 am to 4:30 pm at 25 ± 2°C). Animals were shifted 20 minutes prior to the experimentation room to get accustomed with the test environment. Trials were recorded via videocam, and results were obtained to reveal the final conclusions [15–17].

### 2.7. Morris Water Maze Test

To evaluate 3D spatial recalling memory of the animals under observations, an authentic and sensitive assay “Morris water maze test” was carried out [13].

The apparatus used for this test has a round pool with a 120 cm diameter and a 60 cm of depth. The pool is divided into 4 hypothetical quadrants North, West, South, and East. The experiment began on day 28, and 5 trials were executed each day. The mouse was given 1 min to find out the hidden platform with a 10 min interval between the two successive trials. The usual time essential for a mouse to reach at the platform was recorded, and an average of 5 trials was presumed as the escape latency of the animal for that day. On the 32^nd^ day of intervention, a probe test was executed when there was no platform, with a different release position. The spatial memory of the mouse was estimated by computing the time spent by the mouse in the quadrant in which the platform was formerly located. The number of crossings across the earlier platform position was also found out [18, 19].

### 2.8. Novel Object Recognition Memory Test

The novel object recognition memory test is an additional procedure to describe and evaluate the memory of the rodents. The entire methodology was performed in a specific designed box of dimensions about 25 cm × 25 cm × 25 cm at daytime (25 ± 2°C). It was composed of quarter phases: (1) prehabituation, (2) habituation, (3) training, and (4) testing. All the mice that need to be tested were brought inside the testing room 30-35 min earlier than the commencement of the trial on the 1^st^ day, to get familiarized with the surroundings. They were kept to freely explore the testing box with objects for a 5 min period [20]. The rodents were habituated inside the box for 20 min on days 2 and 3. On day 4, each mouse was brought out to a trial of training accompanied by a trial of testing. In the training trial, 2 objects were located oppositely to each other inside the box at a similar space from the adjacent corner [21]. The rodents were allowed to observe the objects for 10 min and were carried back to their home cages. Subsequently, they were put back to the similar specific investigational box, while this time, one of the two recognized objects was exchanged by a novel/new object [22]. All the performances were videotaped with a camera, and the recognition index was computed with the following formula: recognition test = [time spent with the novel object/time spent with object 1 + novel object] × 100.

### 2.9. Statistical Analysis

All numerical values were expressed as mean ± SEM. One-way ANOVA was used to express the results statistically, and the value of significance was set at *p* < 0.05.

## 3. Results

**Figure 1:**
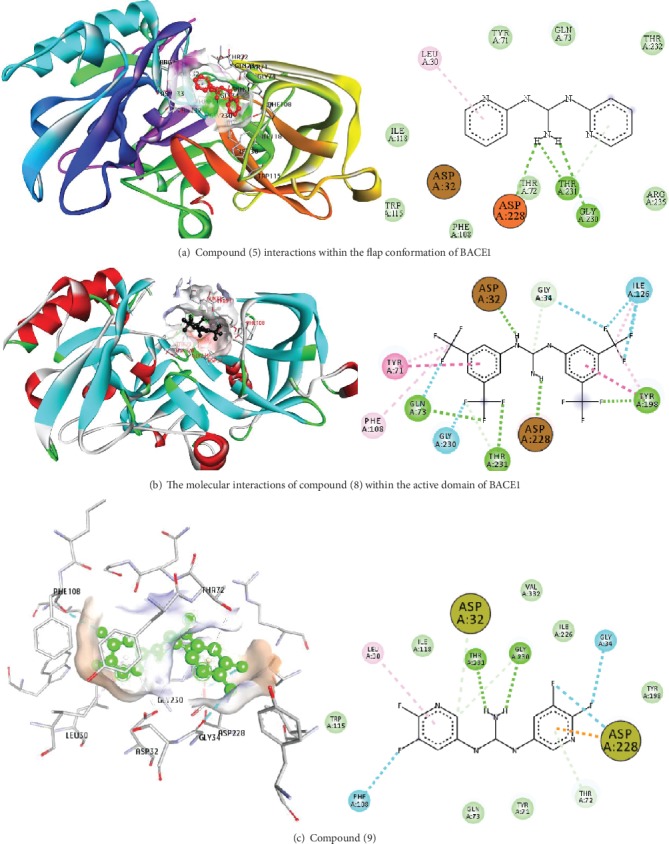
The possible interaction of compounds (5), (8), and (9) within the active domain of BACE1 that completely arrested the catalytic activity of aspartate residues for this enzyme in a closed conformation of the flap region.

**Figure 2:**
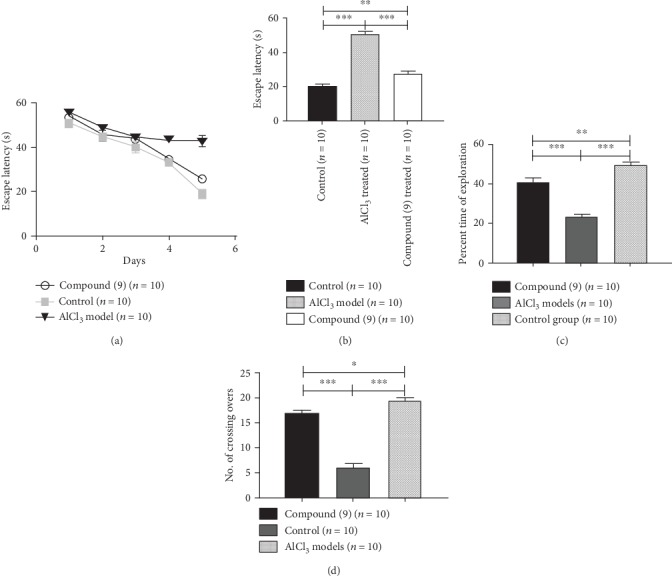
The synthesized compound (9) on the memory/learning behaviors of the mouse models manipulating the available technique of Morris water maze assay. (a) shows the escape latency time period of the mice to arrive at the platform on various trial days of the AlCl_3_-triggered neurotoxicity group, the control group, and the compound (9)-treated group. (b) signifies the analysis results among the three groups in terms of escape latency on the test day. (c) establishes the per hundred total time spent of the mice in the investigations of the target quadrant. (d) demonstrates in what manner each time every mouse crisscrossed the targeted quadrant.

**Figure 3:**
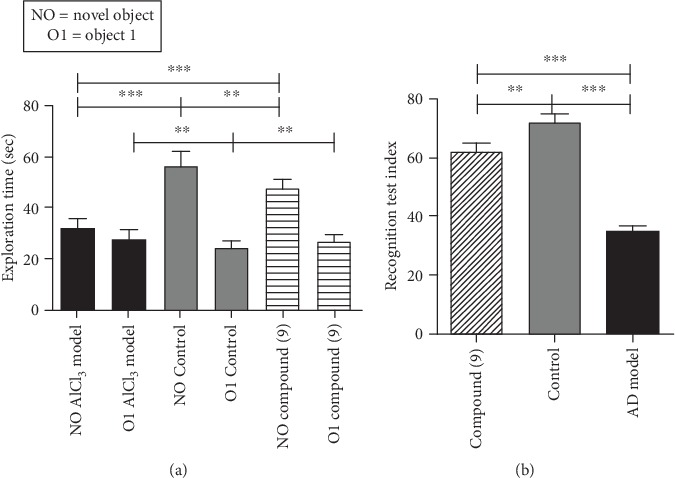
Compound (9) activities have stopped the decline of the 3-D spatial learning/memory of AlCl_3_-induced mouse models at some stage. The average times of explorations and the recognition indices are indicated significant with a *p* < 0.0002 in both cases (one-way ANOVA test). The *f*-ratio value is 10.80; all the results are significant at *p* < 0.05; error bars represent mean ± SEM; the (∗∗) shows less difference while (∗∗∗) shows a statistical significant difference.

This study reported a series of thirteen newly synthesized biaryl guanidine (fluorinated heteroaromatic side chain substituted) ligands intended to inhibit *β*-secretase enzymes. Among them, 1,3-bis(2-fluorophenyl)guanidine (1); 1,3-bis(4-bromonaphthalen-1-yl)guanidine (2); 1,3-bis(2-nitrophenyl)guanidine (3); 1,3-di(pyridin-2-yl)guanidine (4); 1,3-di(quinolin-6-yl)guanidine (5); 1,3-bis(4-fluoronaphthalen-2-yl)guanidine (6); 1,3-di(naphthalen-2-yl)guanidine (7); 1,3-bis(3,5-bis(trifluoromethyl)phenyl)guanidine (8); 1,3-bis(5,6-difluoropyridin-3-yl)guanidine (9); 1,3-bis(4-bromophenyl)guanidine (10); 1,3-bis(4-chloro-2-fluorophenyl)guanidine (11); 1,3-bis(4-fluorophenyl)guanidine (12); and 1,3-bis(3,4-difluorophenyl)guanidine (13) were enlisted. The entire series of the ligands was docked into the active catalyzing pocket of BACE1. Selected docking results were examined, and the maximum values of the compounds fill generally the active catalyzing domain of the dyadic aspartate within the flap region of the BACE1 enzyme.  Figure 1 describes in detail the interaction of the best novel compounds (5), (8), and (9) within the active domain of the flap region of the BACE1 in closed conformation. They were found to inhibit the catalytic activity of the aspartate residues of this enzyme for intended interventions of AD. Among all the synthesized ligands, compound (9) was found to pose 99% BACE1 inhibitory potential (IC_50_ = 97 ± 0.91 nM) within the active pocket of the two key aspartic acids at 500 pmol having the optimum values of all parameters. Compound (10) was found the least effective (35% BACE1 inhibition with IC_50_ = 321 ± 2.15 *μ*M) while the rest of was found in between the highest (9) and the lowest (10) effective compounds.  Table 1 describes in detail chemical structures, spectroscopic analysis, IC_50_, and some important physical/chemical properties while Table 2 summarizes pharmacokinetics and virtually obtained parameters (Figures 1S to 24Srepresented NMR spectra of the newly synthesized compounds available as supplementary material).

The *in vivo* assay “Morris water maze test” explained the aluminum chloride-induced neurotoxicity which is shown by an increase in time of escape latency (50.60 ± 3.70) compared to the control group (20.20 ± 2.01) of mice at day 5. Compound (9) significantly improved the graphical record along with behavior of spatial learning and recalling power (27.72 ± 2.60; *p* < 0.05) when compared with AlCl_3_-induced neurotoxic mice; similarly, an extra time was utilized in exploration of the targeted quadrant (49.60 ± 2.35) while the AD-induced rodents showed23.6 ± 2.01and the treated mice showed a quite better interest in the quadrant lying a stand which on average is41.07 ± 2.5. The AD model crisscrossed the respective quadrant (6.10 ± 1.0) while the control group spanned quite frequently (19.80 ± 1.90). Moreover, the compound (9)-treated group of mice presented the results within 2 designated groups (16.70 ± 1.07) Figure 2.

The novel/new object recognition test showed insignificant variations in the time spent by the rodents in the initial training session. On the testing day, the recognition test index (RTI) was found to be 17.8%, 23.2%, and 62.9%, respectively, for the control, AD model, and AD model-treated mice with our ligand “compound (9)” (*p* < 0.05) as shown in Figure 3. The notable exploration time for the control (64.44 ± 2.50 s), AD model (31.80 ± 2.00 s), and AD model treated with compound (9) (53.80 ± 2.15 s) (*p* < 0.05) was also compared (Figure 2). The exploration time and RTI which were the average times of explorations and the recognition indices have shown critical differences with a *p* < 0.0002 in both cases (one-way ANOVA test). The *f*-ratio value is 10.80; all the results are significant at *p* < 0.05. Newly synthesized compound (9) was found to have stopped the decline of the recognition power of the mice as represented by the results obtained from the *in vivo* assays which were perfectly parallel with our *in vitro* outcomes which were in turn in accordance with the *in silico* results for the BACE1 inhibitory activity.

## 4. Discussion

Aspartic protease BACE1 (*β*-secretase) catalysis is a rate-limiting step for conversion of APP into A*β*42 that aggregated and led to senile plaque formation which is a well-known pathogenesis of AD [1, 21]. The combined effects strengthened the amyloid cascade hypothesis, therefore targeting BACE1 as a therapeutic strategy to stop the progression of AD. According to the literature survey, a lot of work has been done to come up with computationally designed, virtually screened, and chemically synthesized potent BACE1 inhibitors; however, a dire need has been felt recently to develop novel inhibitors to arrest *β*-secretase activity [22]. The present work predicted thirteen ligands as active through virtual screening, which eventually were synthesized as new compounds against *β*-secretase; therefore, compound (9) was found to have better inhibitory activity; it has fluorines attached directly to the side chains of the heteroaromatic ring of the guanidine nucleus [21]. Fluorine attachment imparted remarkable BACE1 inhibitory activity, besides an improved bioavailability at the active domain of the flap region of the BACE1 enzyme. Virtual screening further predicted that the ligands with binary aryl side chains were linked with a wide array of BACE1 inhibitory potentials. Any variation in fluorine moiety positions was found to have resulted in a drastic shift in attachment within the flap region in a closed conformation of BACE1 enzymes [22, 23]. It was revealed that fluorine atoms on the pyridine ring imparted enhanced efficiency (99% BACE1 inhibition; IC_50_ = 97 ± 0.91 nM) and better bioavailability to arrest harmful effects at the specified target domain of BACE1 enzymes.

The Morris water maze behavioral test was employed to analyze the spatial memory in mice and remained valuable to examine the deficits in performances with AD animal models. However, strict infectious agent-free environment is a complex practice and often poses stress to the mice, so this test was also accompanied with another behavioral assay “novel object recognition assay.” The novel object recognition test is based on the acquired instinct of animals to explore the novelty [24, 25]. Moreover, this test did not need any positive or negative stimulus which sometimes negatively affects the results. On the account of all physiologic relevance, and to balance the results of MWMA if there is any deficiency, the NORT has also been carried out [23, 24].

## Figures and Tables

**Table 1:** Detailed information about newly synthesized N,N′-biaryl guanidine derivatives to arrest BACE1 enzymatic activity.

Code	Structure of the compound with name	Spectral detail (NMR & ESI-MS)	Miscellaneous features
1	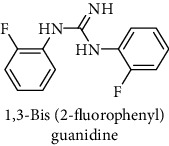	^1^H-NMR (500 MHz, CD_3_OD): *δ* 8.6 (2H, NH, s), 8.2 (1H, NH, s), 7.3 (2H, d, *J* = 7.6 Hz), 7.0 (2H, d, *J* = 7.5 Hz), 6.8 (2H, d, *J* = 9 Hz), 6.7 (2H, d, *J* = 7.5 Hz); ^13^C-NMR (125 MHz, CD_3_OD): *δ* 165.3, 163.3, 139.5 (2C), 135.7, 132.0 (2C), 122.7 (2C), 122.6 (2C), 114.1, 113.9; ^19^F-NMR (470 MHz, CD_3_OD): *δ* -108.06, -125.11; HR-EI MS: *m*/*z* 247.09201; [(*M* + 1)^+^ calculated for C_13_H_11_F_2_N_3_ 247.09210].	55% yield; white solid; mp 130-132°C; IC_50_ = 85 ± 1.84 *μ*M HB_donor 3; HB_acceptor 3; rotatable bonds 4; Ar-Br was used to synthesize; M.Wt = 247.092 g/mol

2	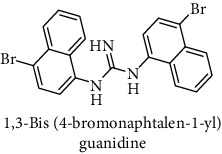	^1^H-NMR (500 MHz, CD_3_OD): *δ* 8.8 (2H, NH, s), 8.2 (2H, d, *J* = 7.8 Hz), 7.9 (2H, d, *J* = 7.1 Hz), 7.8 (3H, d, *J* = 8.1 Hz), 7.4 (4H, m), 6.9 (2H, d, *J* = 7 Hz); ^13^C-NMR (125 MHz, CD_3_OD): *δ* 161.3, 143.2 (2C), 131.9 (2C), 131.1 (2C), 129.3 (2C), 128.7 (2C), 126.8 (2C), 126.3 (2C), 121.6 (2C), 114.5 (2C), 110.9 (2C); HR-EI MS: *m*/*z* 466.96318; [(*M* + 1)^+^ calculated for C_21_H_15_Br_2_N_3_ 466.96327].	79% yield; white solid; mp 128-130°C; IC_50_ = 235 ± 1.90 *μ*M; HB_donor 4; HB_acceptor 1; rotatable bonds 3; Ar-Br was used to synthesize; M.Wt = 466.963 g/mol

3	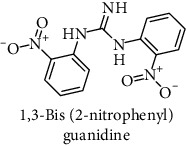	^1^H-NMR (500 MHz, CD_3_OD): *δ* 8.6 (2H, NH, s), 8.3 (1H, NH, s), 7.7 (3H, m), 7.4 (3H, m), 7.0 (2H, m); ^13^C-NMR (125 MHz, CD_3_OD): *δ* 158.5, 139.7 (2C), 132.5 (2C), 131.0 (2C), 126.4 (2C), 123.0 (2C), 111.0 (2C); HREI MS: *m*/*z* 301.8121; [(*M* + 1)^+^ calculated for C_13_H_11_N_5_O_4_ 301.8110].	92% yield; yellow solid; mp 136-138°C; IC_50_ = 259 ± 2.50 *μ*M; HB_donor 5; HB_acceptor 3; rotatable bonds 6; Ar-Br was used to synthesize; M.Wt = 301.881 g/mol

4	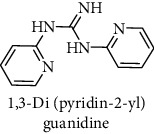	^1^H-NMR (500 MHz, CD_3_OD): *δ* 8.2 (2H, s), 7.6 (3H, d, *J* = 7.5 Hz), 6.9 (3H, t, *J* = 7.8 Hz); ^13^C-NMR (125 MHz, CD_3_OD): *δ* 160.8, 159.8 (2C), 149.2 (2C), 139.7 (2C), 119.4 (2), 111.9 (2C); HREI MS: *m*/*z* 213.10133; [(*M* + 1)^+^ calculated for C_11_H_11_N_5_ 213.10145].	88% yield; white solid; mp 140-142°C; IC_50_ = 9 ± 0.05 *μ*M; HB_donor 3; HB_acceptor 3; rotatable bonds 4; Ar-Br was used to synthesize; M.Wt = 213.101 g/mol

5	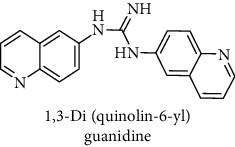	^1^H-NMR (500 MHz, CD_3_OD): *δ* 9.8 (1H, NH, s), 8.6 (2H, d, *J* = 8 Hz), 8.3 (1H, NH, s), 8.1 (1H, NH, S), 8.0 (1H, s), 7.7 (1H, s), 7.5 (2H, d, *J* = 5), 7.3 (6H, m). ^13^C-NMR (125 MHz, CD_3_OD): *δ* 161.6, 146 (2C), 143.2 (2C), 138.3 (2C), 133.4 (2C), 132.6 (2C), 130.3 (2C), 124.9 (2C), 123.5 (2C), 121.7 (2C); HREI MS: *m*/*z* 313.13265; [(*M* + 1)^+^ calculated for C_19_H_15_N_5_ 313.13275].	90% yield; white solid; mp 133-134°C; IC_50_ = 91 ± 0.19 nM; HB_donor 3; HB_acceptor 3; rotatable bonds 4; Ar-Br was used to synthesize; M.Wt = 313.132 g/mol

6	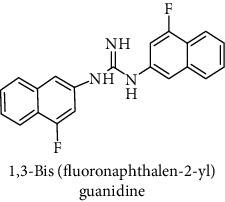	^1^H-NMR (500 MHz, CD_3_OD): *δ* 9.2 (2H, NH, s), 8.2 (2H, m), 7.9 (2H, d, *J* = 8.5 Hz), 7.8 (2H, m), 7.6 (1H, NH, S), 7.5 (2H, dt, *J* = 7 Hz), 7.4 (1H, m), 7.2 (2H, m), 6.8 (1H, S); ^13^C-NMR (125 MHz, CD_3_OD): *δ* 161.5, 160.4 (2C), 139.3 (2C), 134.5 (2C), 129.3 (2C), 127.6 (2C), 127.1 (2C), 124.6 (2C), 123.1 (2C), 118.0 (2C) 105.6 (2C); ^19^F-NMR (470 MHz, CD_3_OD): *δ* -75.2; HREI MS: *m*/*z* 347.12329; [(*M* + 1)^+^ calculated for C_21_H_15_F_2_N_3_ 347.12340].	63% yield; white solid; mp 126-128°C; IC_50_ = 102 ± 0.90 nM; HB_donor 3; HB_acceptor 3; rotatable bonds 4; Ar-Br was used to synthesize; M.Wt = 347.123 g/mol

7	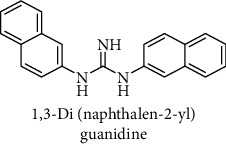	^1^H-NMR (500 MHz, CD_3_OD): *δ* 9.2 (2H, NH, s), 8.2 (4H, m), 7.94 (1H, q, *J* = 5, 5.5 Hz), 7.8 (2H, tt, *J* = 7, 7.5 Hz), 7.8 (3H, m), 7.4 (2H, q, *J* = 8.5 Hz), 6.8 (1H, NH, s); ^13^C-NMR (125 MHz, CD_3_OD): *δ* 161.9, 137.5 (2C), 135.2 (4C), 134.5 (2C), 133.2 (2C), 132.0 (2C), 129.5 (2C), 126.1 (2C), 121.7 (2C), 116.4 (2C); HREI MS: *m*/*z* 311.14215; [(*M* + 1)^+^ calculated for C_21_H_17_N_3_ 311.14225].	91% yield; white solid; mp 146-148°C; IC_50_ = 159 ± 1.10 *μ*M; HB_donor 3; HB_acceptor 1; rotatable bonds 4; Ar-Br was used to synthesize; M.Wt = 311.142 g/mol

8	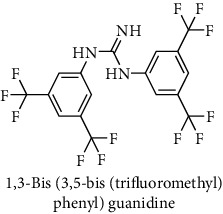	^1^H-NMR (500 MHz, CD_3_OD): *δ* 8.9 (2H, NH, s), 7.3 (3H, s), 5.7 (4H, s); ^13^C-NMR (125 MHz, CD_3_OD): *δ* 161.3, 146.3 (2C), 134.7 (4C, d, *J* = 5.1 Hz), 131.4 (2C), 125.8 (4C, d, *J* = 271.3 Hz), 123.7 (2C, d, *J* = 34.6 Hz), 115.7 (2C); ^19^F-NMR (470 MHz, CD_3_OD): *δ* -61.8, -61.5; HREI MS: *m*/*z* 483.06039; [(*M* + 1)^+^ calculated for C_17_H_9_F_12_N_3_ 483.06049].	68% yield; yellowish solid; mp 76-78°C; IC_50_ = 229 ± 2.10 *μ*M; HB_donor 3; HB_acceptor 13; rotatable bonds 8; Ar-I was used to synthesize; M.Wt = 483.060 g/mol

9	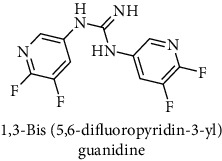	^1^H-NMR (500 MHz, CD_3_OD): *δ* 8.7 (2H, NH, s), 8.3 (2H, s), 7.4 (1H, s), 7.3 (2H, t, *J* = 10.5 Hz); ^13^C-NMR (125 MHz, CD_3_OD): *δ* 166.9 (2C), 158.5, 146.3 (2C), 143.2 (2C), 135.7 (2C), 121.5 (2C); HREI MS: *m*/*z* 285.06376; [(*M* + 1)^+^ calculated for C_11_H_7_F_4_N_5_ 285.06386].	61% yield, whitish yellow solid; mp 128-130°C; IC_50_ = 97 ± 0.91 nM; HB_donor 3; HB_acceptor 7; rotatable bonds 4; Ar-Br was used to synthesize; M.Wt = 285.063 g/mol

10	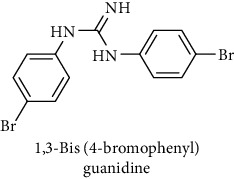	^1^H-NMR (500 MHz, CD_3_OD): *δ* 7.5 (2H, NH, s), 7.3 (4H, d, *J* = 8.5 Hz), 7.3 (4H, d, *J* = 8.5 Hz), 6.7 (1H, NH, s); ^13^C-NMR (125 MHz, CD_3_OD): *δ* 163.3, 153.4, 133.8 (4C), 121.1 (4C); HREI MS: *m*/*z* 368.93187; [(*M* + 1)^+^ calculated for C_13_H_11_Br_2_N_3_ 368.93197].	72% yield; white solid; mp 250-252°C; IC_50_ = 321 ± 2.15 *μ*M; HB_donor 3; HB_acceptor 1; rotatable bonds 4; Ar-Br was used to synthesize; M.Wt = 368.931 g/mol

11	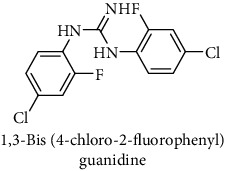	^1^H-NMR (500 MHz, CD_3_OD): *δ* 8.4 (2H, NH, s) 7.9 (2H, dd, *J* = 2, 10.5 Hz), 7.8 (2H, d, *J* = 7 Hz), 7.6 (2H, t, *J* = 9 Hz), 6.2 (1H, s); ^13^C-NMR (125 MHz, CD_3_OD): *δ* 159.1 (2C), 130.3 (2C), 126.2 (2C), 121.2 (2C), HREI MS: *m*/*z* 315.01425; [(*M* + 1)^+^ calculated for C_13_H_9_Cl_2_F_2_N_3_ 315.01416].	69% yield; white solid; mp 160-162°C; IC_50_ = 172 ± 1.50 *μ*M; HB_donor 3; HB_acceptor 3; rotatable bonds 4; Ar-I was used to synthesize; M.Wt = 315.014 g/mol

12	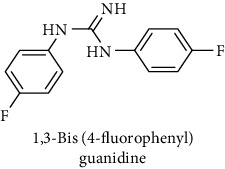	^1^H-NMR (500 MHz, CD_3_OD): *δ* 7.7 (2H, NH, s), 7.1 (4H, t, *J* = 8 Hz), 7.0 (5H, t, *J* = 8.5 Hz), ^13^C-NMR *δ* (125 MHz, CD_3_OD) 161.3, 159.47 (2C), 127.6 (2C), 127.1 (2C), 126.7 (2C), 121.5 (2C), 116.3 (2C); ^19^F-NMR (470 MHz, CD_3_OD): *δ* -123.4, -123.1; HREI MS: *m*/*z* 247.11200; [(*M* + 1)^+^ calculated for C_13_H_11_F_2_N_3_ 247.11210].	71% yield; white solid; mp 146-148°C; IC_50_ = 103 ± 0.95 *μ*M; HB_donor 3; HB_acceptor 3; rotatable bonds 4; Ar-Br was used to synthesize; M.Wt = 247.112 g/mol

13	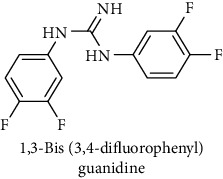	^1^H-NMR (500 MHz, CD_3_OD): *δ* 8.4 (2H, NH, s) 7.6 (2H, t, *J* = 7 Hz), 7.3 (3H, t, *J* = 7.8 Hz), 7.0 (2H, m); ^13^C-NMR (125 MHz, CD_3_OD) *δ* 161.9 (2C), 158.8, 133.9 (2C), 130.1 (2C), 130.0, 126.4, 124.6, 117.1, 116.8, 108.5; ^19^F-NMR (470 MHz, CD_3_OD): *δ* -108.0, -125.1; HR-EI MS: *m*/*z* 283.07316; [(*M* + 1)^+^ calculated for C_13_H_9_F_4_N_3_ 283.07326].	56% yield; white solid; mp150-152; IC_50_ = 71 ± 0.80 *μ*M; HB_donor 3; HB_acceptor 5; rotatable bonds 4; Ar-Br was used to synthesize; M.Wt = 283.073 g/mol

Note: NMR: nuclear magnetic resonance; ESI-MS: electrospray ionization-mass spectrometry; mp: melting point; HB: hydrogen bonding; M. Wt: molecular weight.

**Table 2:** Represents the pharmacokinetics and virtually obtained parameters of newly synthesized biaryl guanidine derivatives.

Code	Log *P*_o/w_ (iLOGP) [26]	Log *S* [27]	Pharmacokinetics		Drug likeness					
			GI absorption	Glycoprotein substrate	Lipinski [28]	Ghose [29]	Veber [30]	Egan [30]	Muegge [30]	BA-score [28]
1	2.06	-3.35	High	Yes	Yes; 0 violation	Yes	Yes	Yes	Yes	0.55
2	3.25	-3.80	High	No	Yes; 0 violation	Yes	Yes	Yes	Yes	-5.77
3	1.26	-3.80	Low	No	Yes; 0 violation	Yes	Yes	Yes	Yes	0.55
4	1.64	-2.5	High	No	Yes; 0 violation	Yes	Yes	Yes	Yes	0.55
5	2.30	-4.09	High	Yes	Yes; 0 violation	Yes	Yes	Yes	Yes	
6	2.65	-5.62	High	No	Yes; 1 violation: MLOGP > 4.15	No; 1 violation: WLOGP > 5.6	Yes	No; 1 violation: WLOGP > 5.88	No; 1 violation: XLOGP3 > 5	0.55
7	2.44	-5.32	High	Yes	Yes; 1 violation: MLOGP > 4.15	Yes	Yes	Yes	No; 1 violation: XLOGP3 > 5	0.55
8	2.33	-6.43	Low	No	Yes; 1 violation: MLOGP > 4.15	No; 2 violations: MW > 480, WLOGP > 5.6	Yes	No; 1 violation: WLOGP > 5.88	No; 2 violations: XLOGP3 > 5, H − acc > 10	0.55
9	1.76	-2.73	High	Yes	Yes; 0 violation	Yes	Yes	Yes	Yes	0.55
10	2.46	-4.85	High	Yes	Yes; 0 violation	Yes	Yes	Yes	Yes	0.55
11	2.47	-4.52	High	Yes	Yes; 0 violation	Yes	Yes	Yes	Yes	0.55
12	1.91	-3.35	High	Yes	Yes; 0 violation	Yes	Yes	Yes	Yes	0.55
13	1.95	-3.65	High	Yes	Yes; 1 violation: MLOGP > 4.15	Yes	Yes	Yes	Yes	0.55

The pharmacokinetics and all the virtually obtained parameters of biaryl guanidine base ligands under studies. BA: bioavailability; GI: gastrointestinal; GP: glycoprotein; HB: hydrogen bonding; Mol_Wt: molecular weight. iLOGP is the n-octanol/water partition coefficient, Log *S* represents the experimental aqueous solubilities of the synthesized compounds, and similarly MLOGP (Moriguchi octanol-water partition coefficient) is the calculated partition coefficient of the ligands under studies.

## Data Availability

The present data regarding synthesis of compounds was generated in the laboratory of Prof. Dr. Borhan Babak (Department of Chemistry, Michigan State University, East Lansing, Michigan 48824, USA) and would be available on demand from the mentor. However, animal studies and molecular docking were conducted at the Department of Pharmacy, COMSATS University Islamabad, Pakistan, after getting permission from the Departmental Review Committee (Ref. # PHM.Eth/CS-M01/18-001 dated 05/2018). However, FRET assays were performed at Rizvanov's laboratory (KFU, Russia).

## Supplementary Materials

Supplementary Materials Figure 1S: ^1^H-NMR spectrum of compound (1). Figure 2S: ^13^C-NMR spectrum of compound (1). Figure 3S: ^19^F-NMR spectrum of compound (1). Figure 4S: ^1^H-NMR spectrum of compound (2). Figure 5S: ^1^H-NMR spectrum of compound (3). Figure 6S: ^13^C-NMR spectrum of compound (3). Figure 7S: ^1^H-NMR spectrum of compound (4). Figure 8S: ^1^H-NMR spectrum of compound (5). Figure 9S: ^1^H-NMR spectrum of compound (6). Figure 10S: ^19^F-NMR spectrum of compound (6). Figure 11S: ^1^H-NMR spectrum of compound (7). Figure 12S: ^13^C-NMR spectrum of compound (7). Figure 13S: ^1^H-NMR spectrum of compound (8). Figure 14S: ^19^F-NMR spectrum of compound (8). Figure 15S: ^1^H-NMR spectrum of compound (9). Figure 16S: ^13^C-NMR spectrum of compound (9). Figure 17S: ^1^H-NMR spectrum of compound (10). Figure 18S: ^13^C-NMR spectrum of compound (10). Figure 19S: ^1^H-NMR spectrum of compound (11). Figure 20S: ^1^H-NMR spectrum of compound (12). Figure 21S: ^19^F-NMR spectrum of compound (12). Figure 22S: ^1^H-NMR spectrum of compound (13). Figure 23S: ^13^C-NMR spectrum of compound (13). Figure 24S: ^19^F-NMR spectrum of compound (13).
