# Supplementary material for: Biological Evaluation of Newly Synthesized Biaryl Guanidine Derivatives to Arrest β-Secretase Enzymatic Activity Involved in Alzheimer's Disease
Source: Biomed Res Int. 2020 May 11;2020:8934289. doi: 10.1155/2020/8934289 (PMC7238388; doi:10.1155/2020/8934289)

**Supporting Information as the Representative NMRs Spectra and Ethical Clearance Form**

**Biological Evaluation of Newly Synthesized Bi-aryl Guanidine Derivatives to Arrest β-secretases Enzymatic Activity Involved in Alzheimer’s Disease**

**Sayyad Ali,^1, 2^ Muhammad Hassham Hassan Bin Asad,^1, 3^ Fahad Khan,^4^ Ghulam Murtaza, ^5^ Albert A Rizvanov,^3^ Jamshed Iqbal,^1^ Borhan Babak,^2^ and Izhar Hussain,^1^**

*^1^ Department of Pharmacy, COMSATS University Islamabad, Abbottabad campus, 22060, Pakistan*

*^2^ Department of Chemistry, Michigan State University, East Lansing, Michigan 48824, U.S.A*

*^3^ Department of Genetics, Institute of Fundamental Medicine and Biology, Kazan Federal University, 420021, Russia*

*^4^ School of Packaging, Michigan state University, East Lansing, Michigan, 48824-1223, U.S.A*

Correspondence should be addressed to Dr. Muhammad Hassham Hassan Bin Asad; hasshamasad@yahoo.com and Izhar Hussain; izharhussain@cuiatd.edu.pk


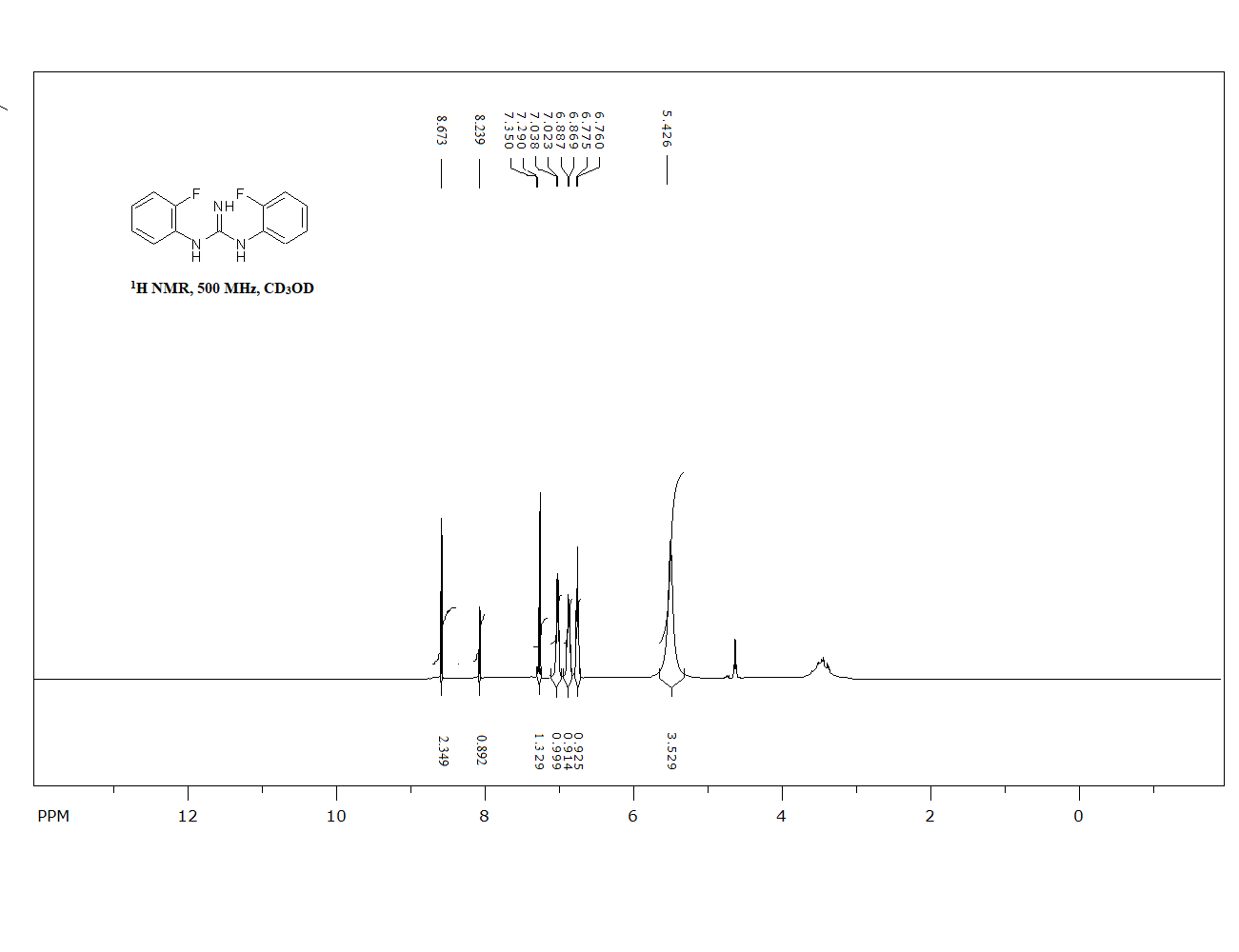


F_IGURE_ 1S: ^1^H NMR spectrum of the compound 1


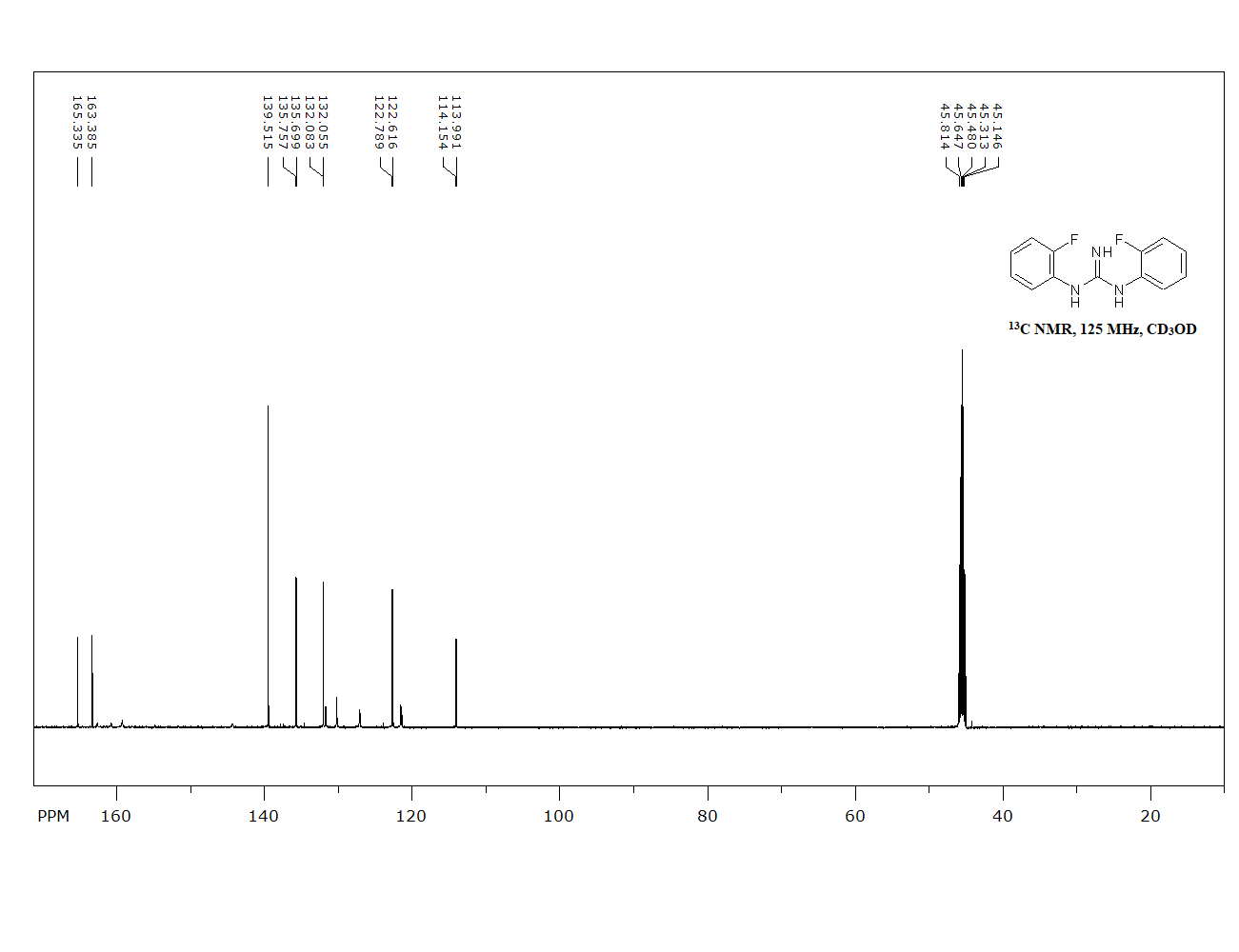


F_IGURE_ 2S: ^13^C-NMR spectrum of the compound 1


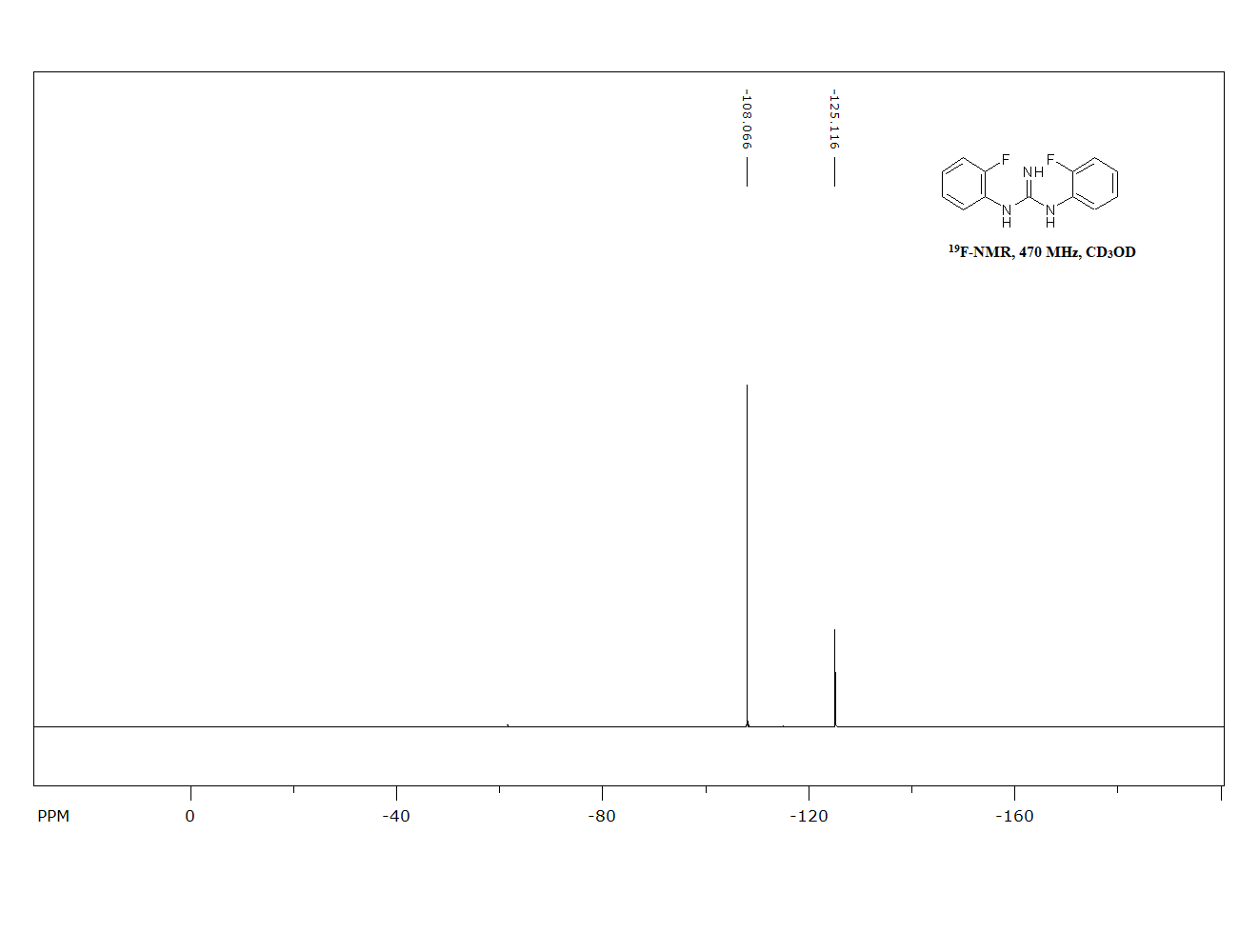


F_IGURE_ 3S: ^19^F-NMR spectrum of the compound 1


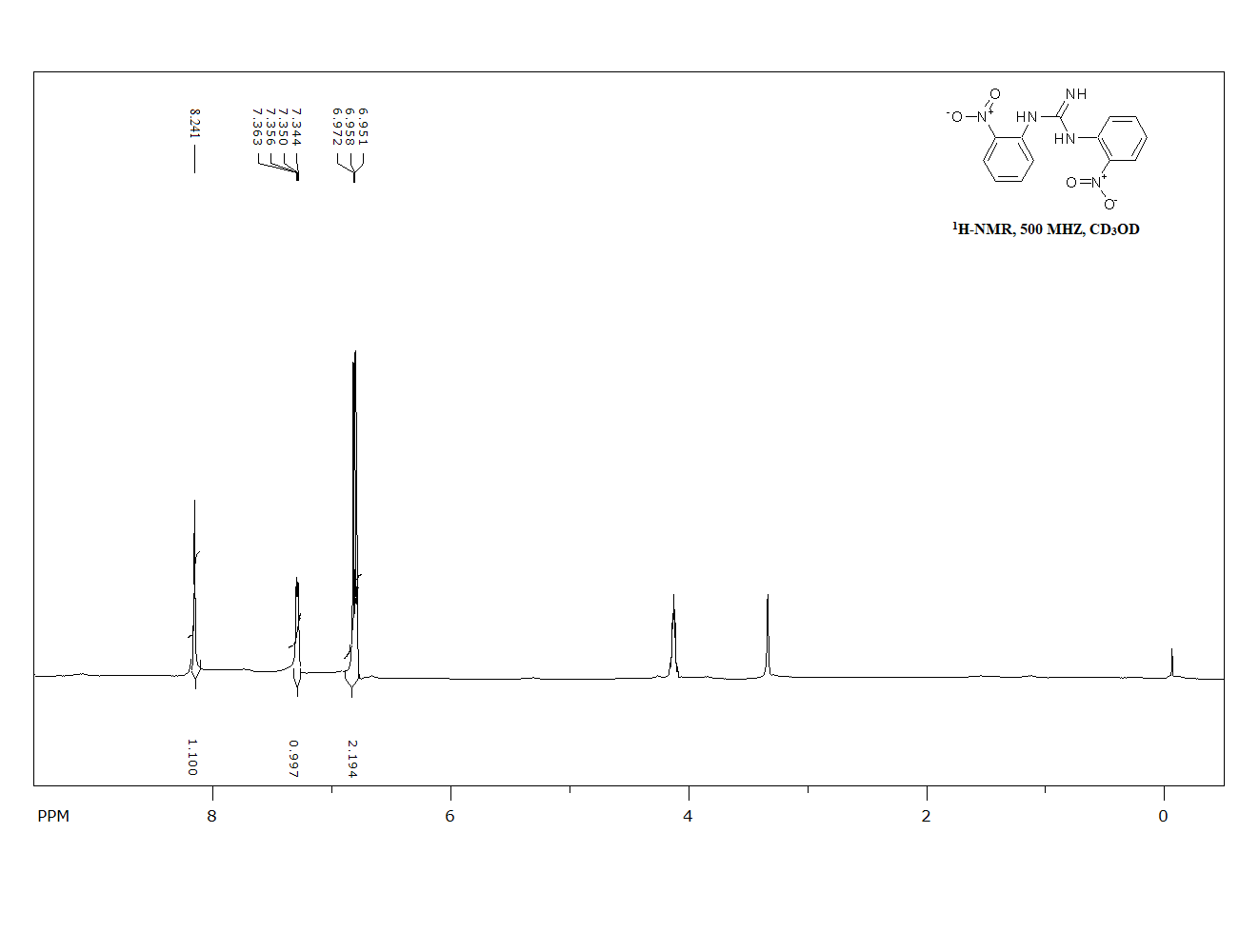


F_IGURE_ 4S: ^1^H-NMR spectrum of the compound 2


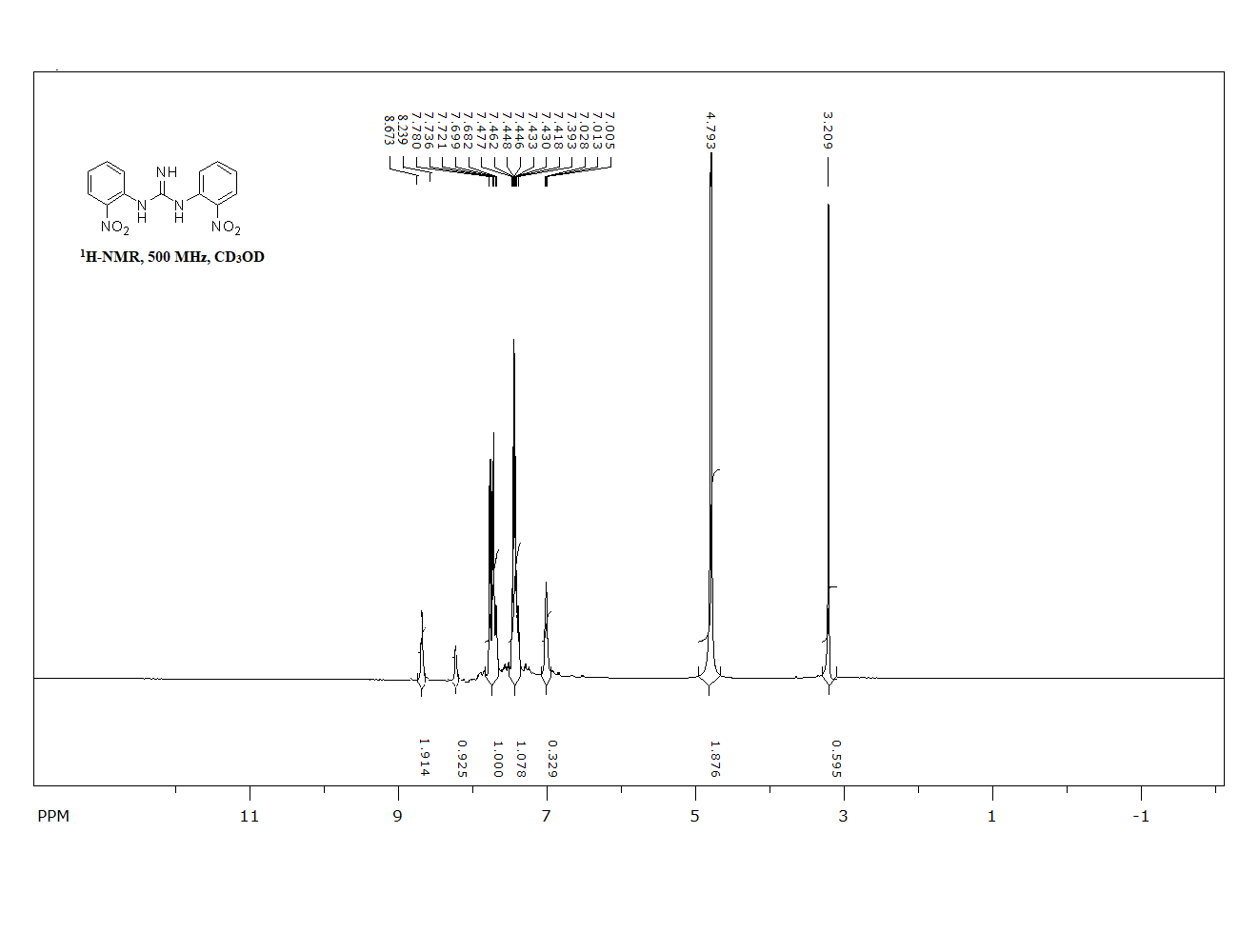


F_IGURE_ 5S: ^1^H-NMR spectrum of the compound 3


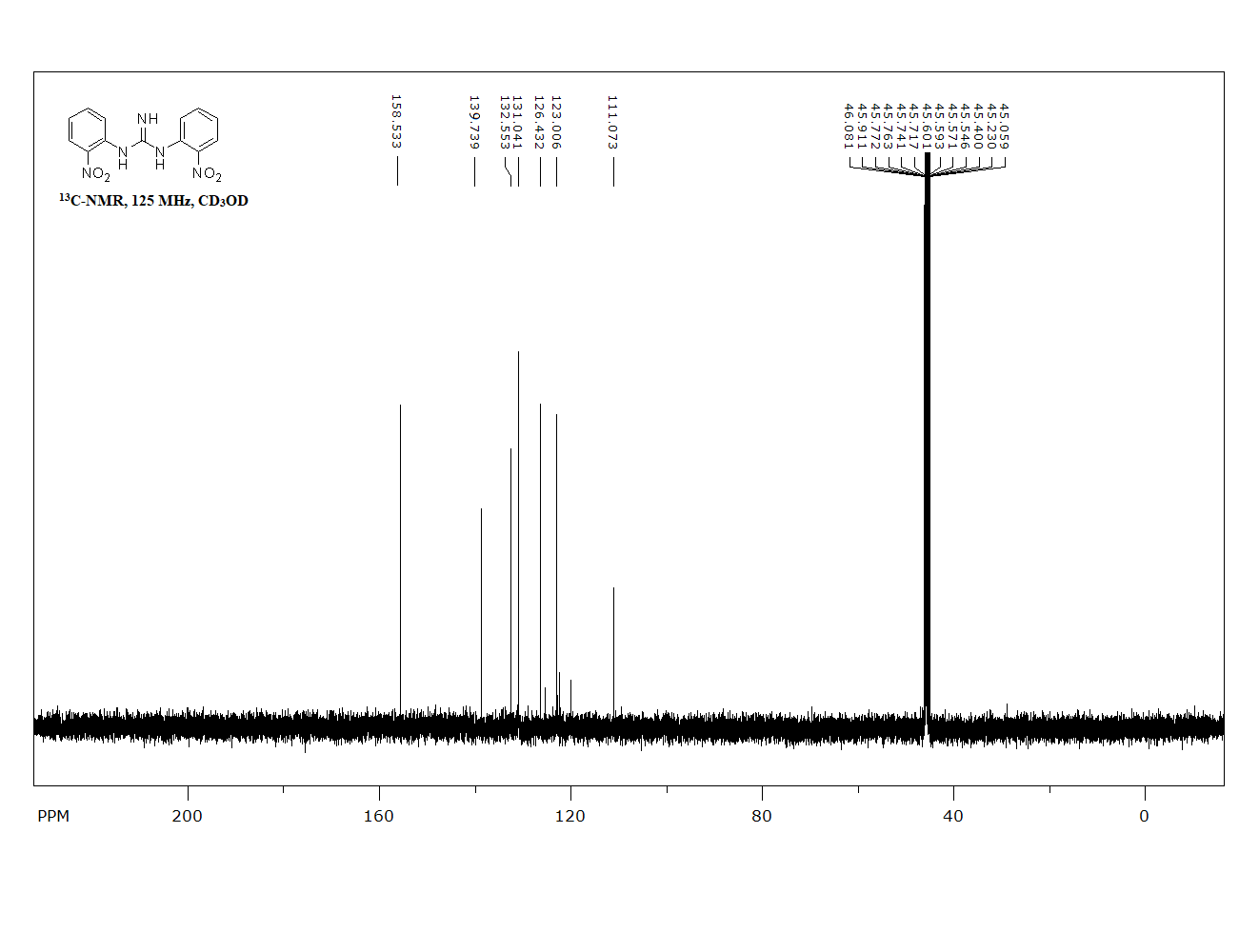


F_IGURE_ 6S: ^13^C-NMR spectrum of the compound 3


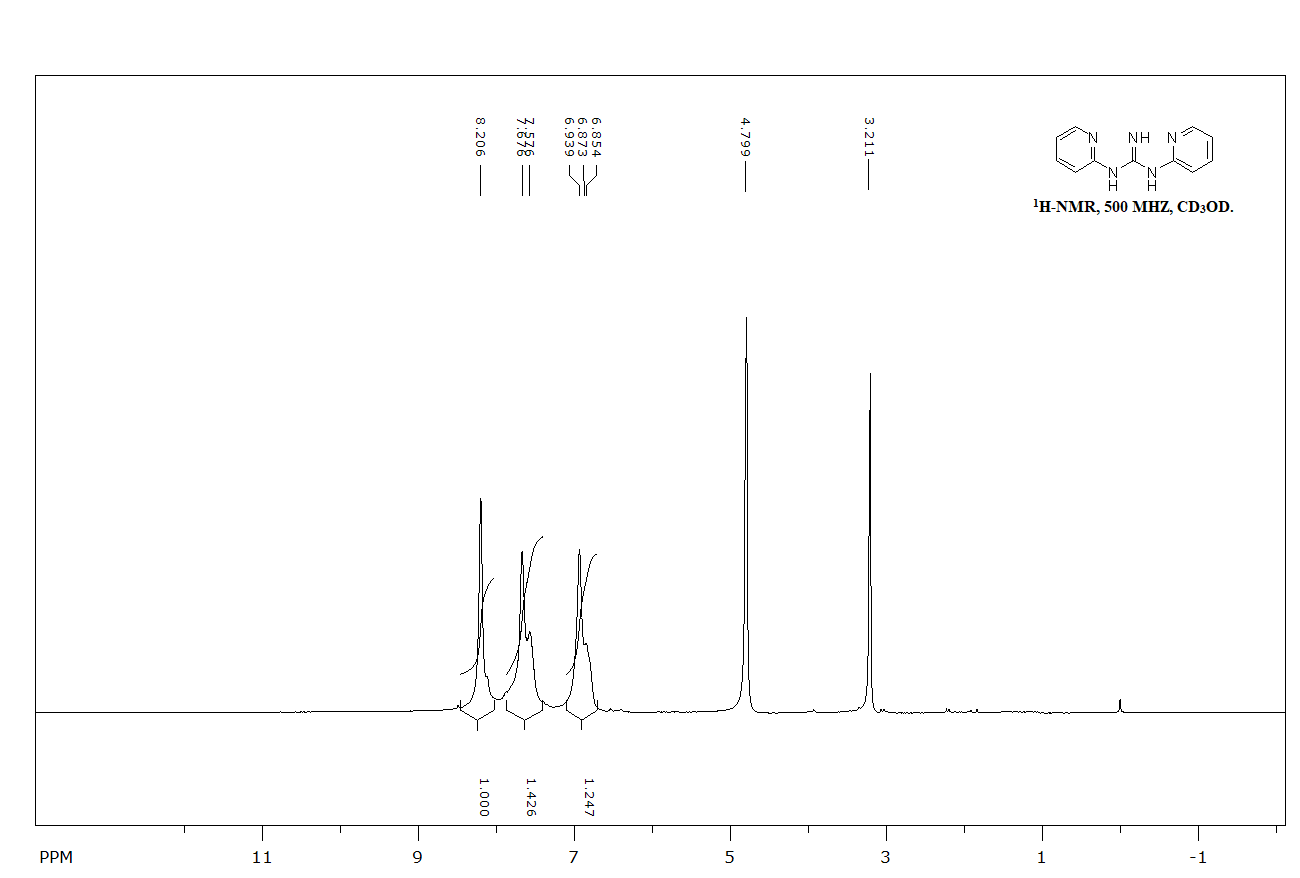


F_IGURE_ 7S: ^1^H-NMR spectrum of the compound 4


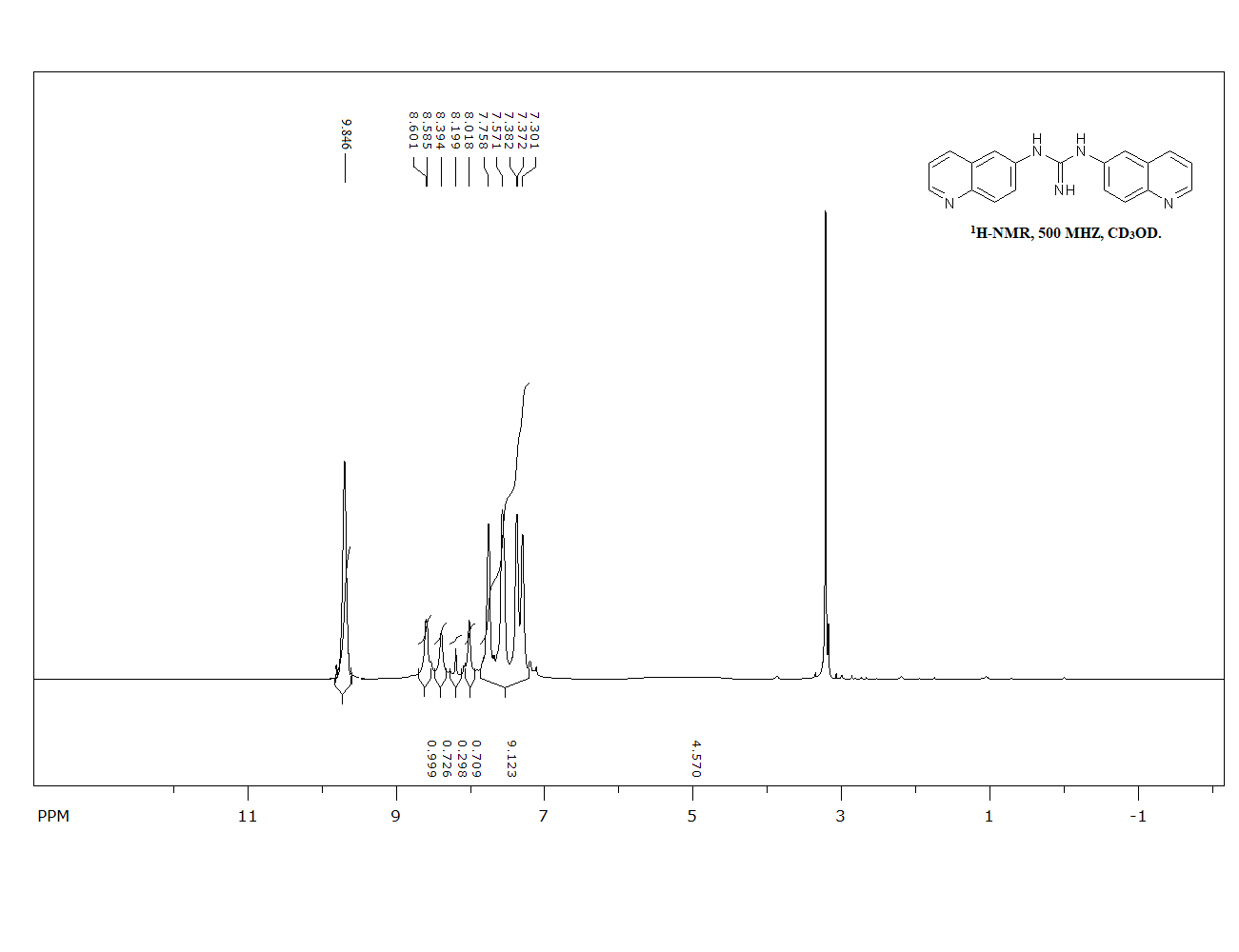


F_IGURE_ 8S: ^1^H-NMR spectrum of the compound 5


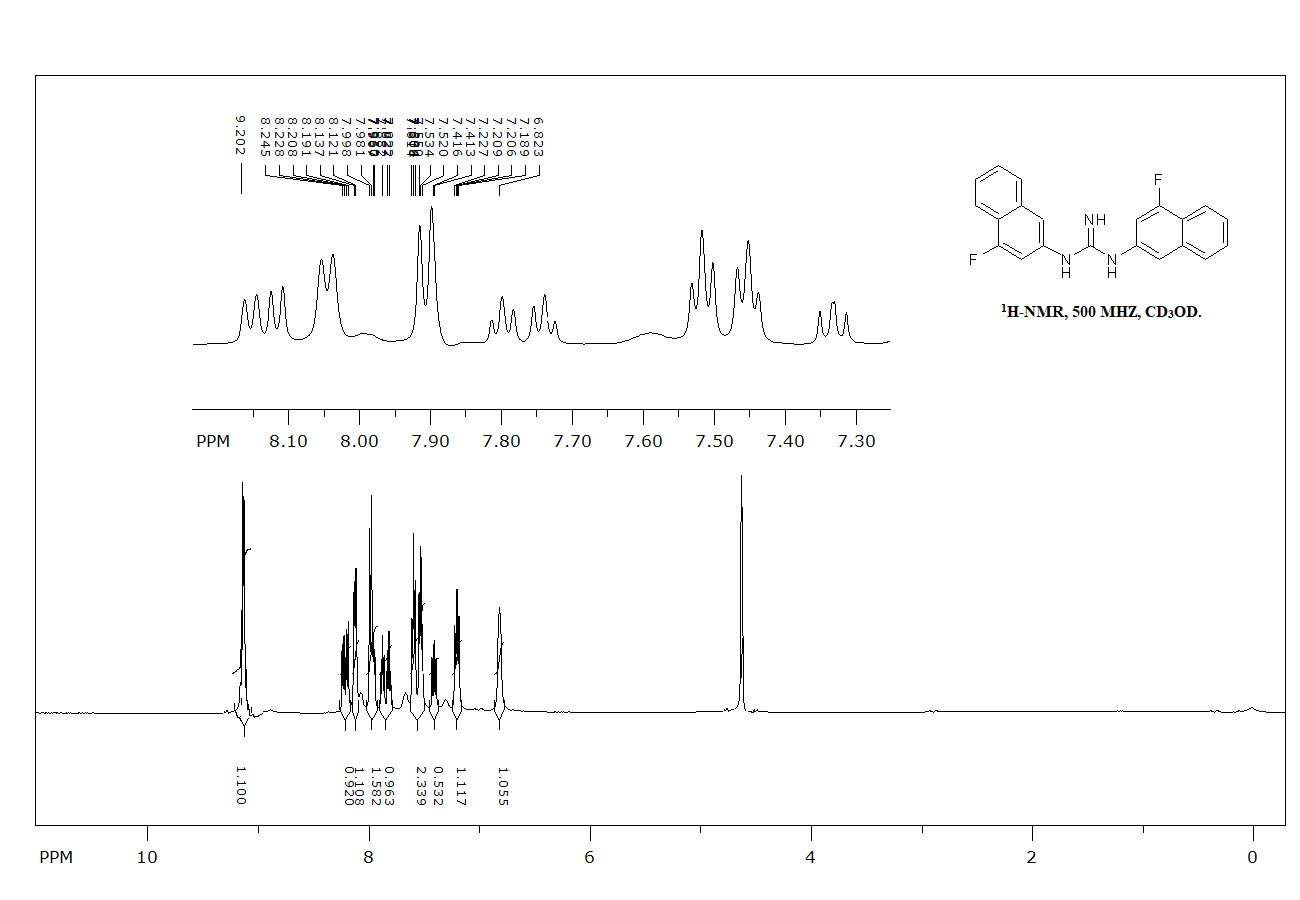


F_IGURE_ 9S: ^1^H-NMR spectrum of the compound 6


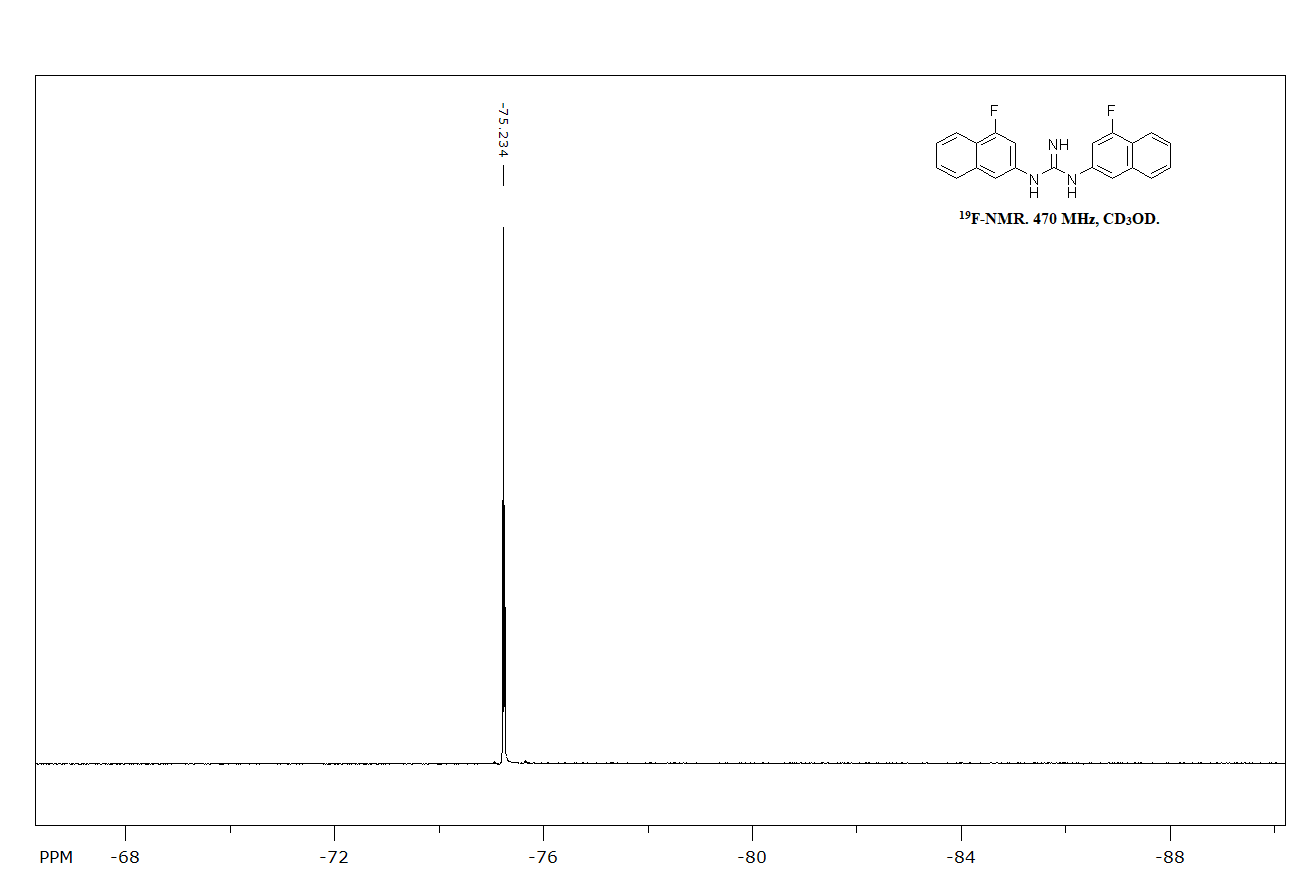


Compound 6 ^19^F-NMR

F_IGURE_ 10S: ^19^F-NMR spectrum of the compound 6


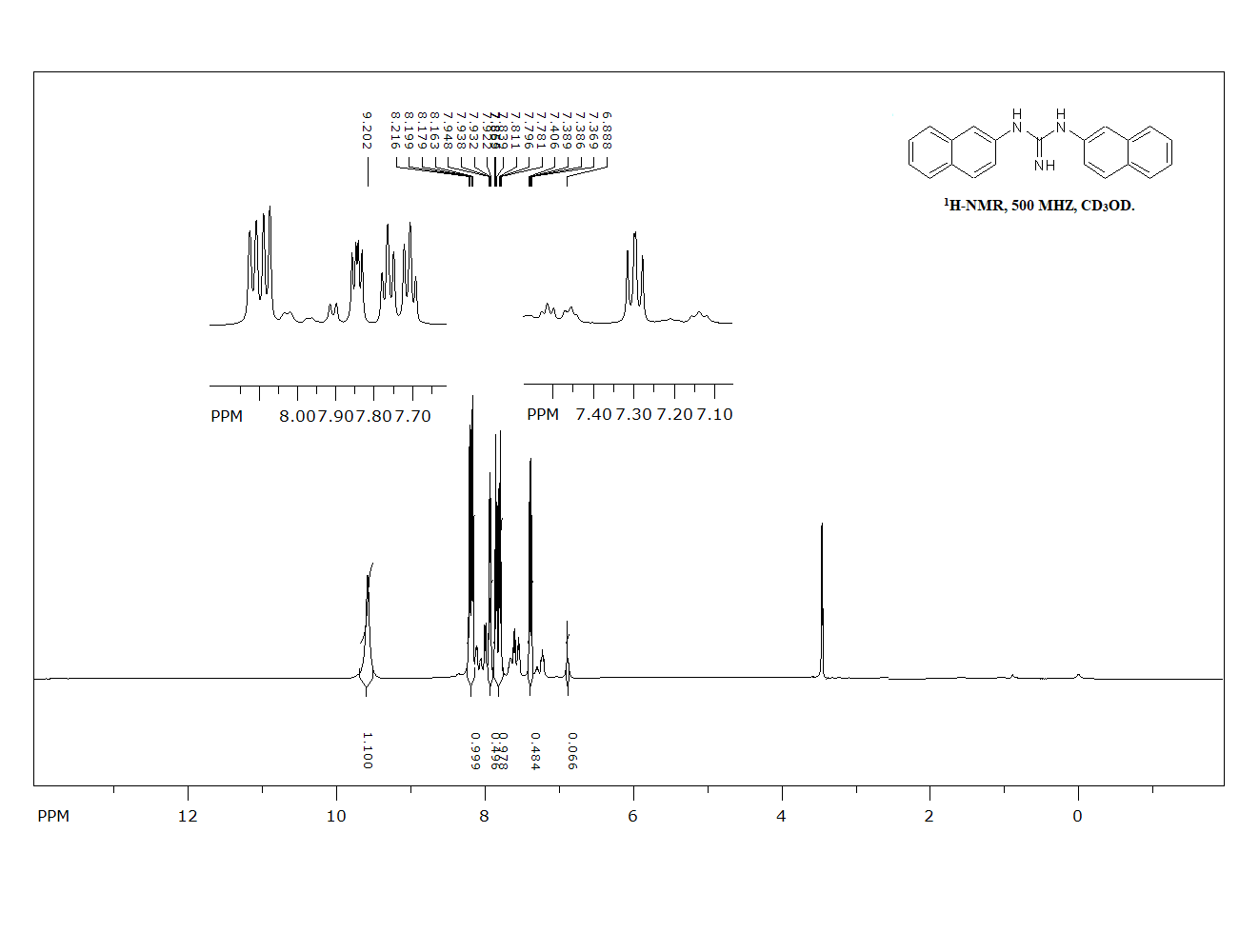


F_IGURE_ 11S: ^1^H-NMR spectrum of the compound 7


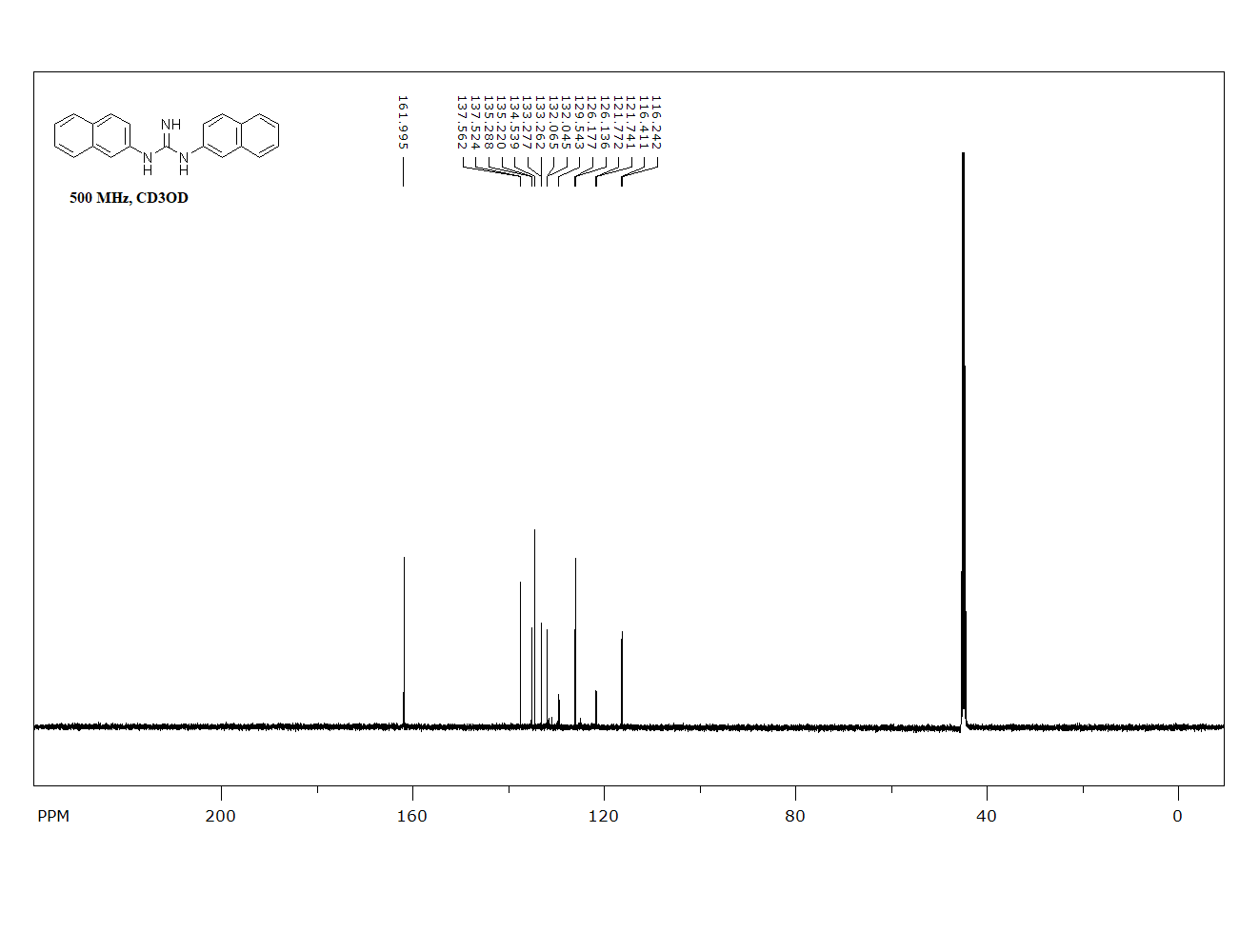


F_IGURE_ 12S: ^13^C-NMR spectrum of the compound 7


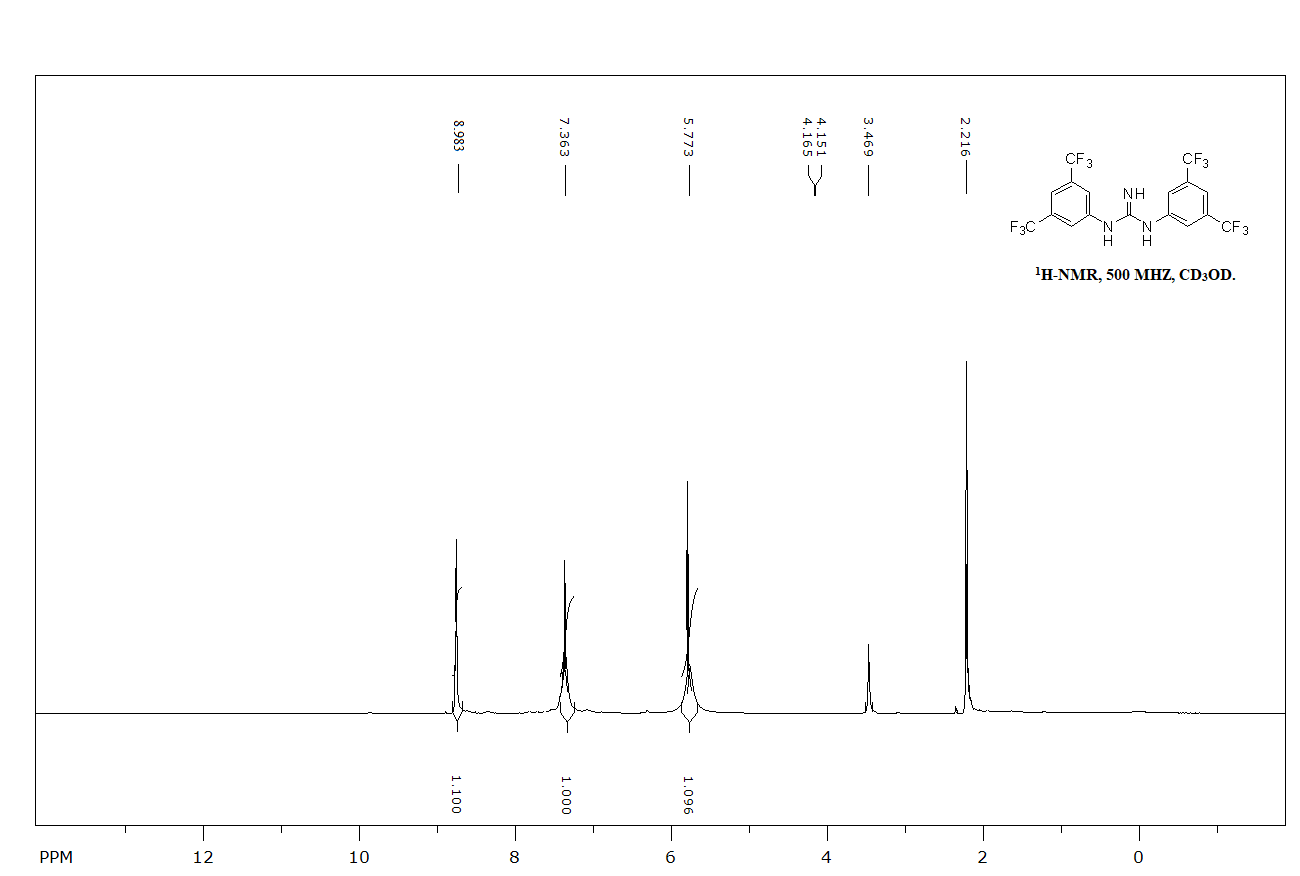


F_IGURE_ 13S: ^1^H-NMR spectrum of the compound 8


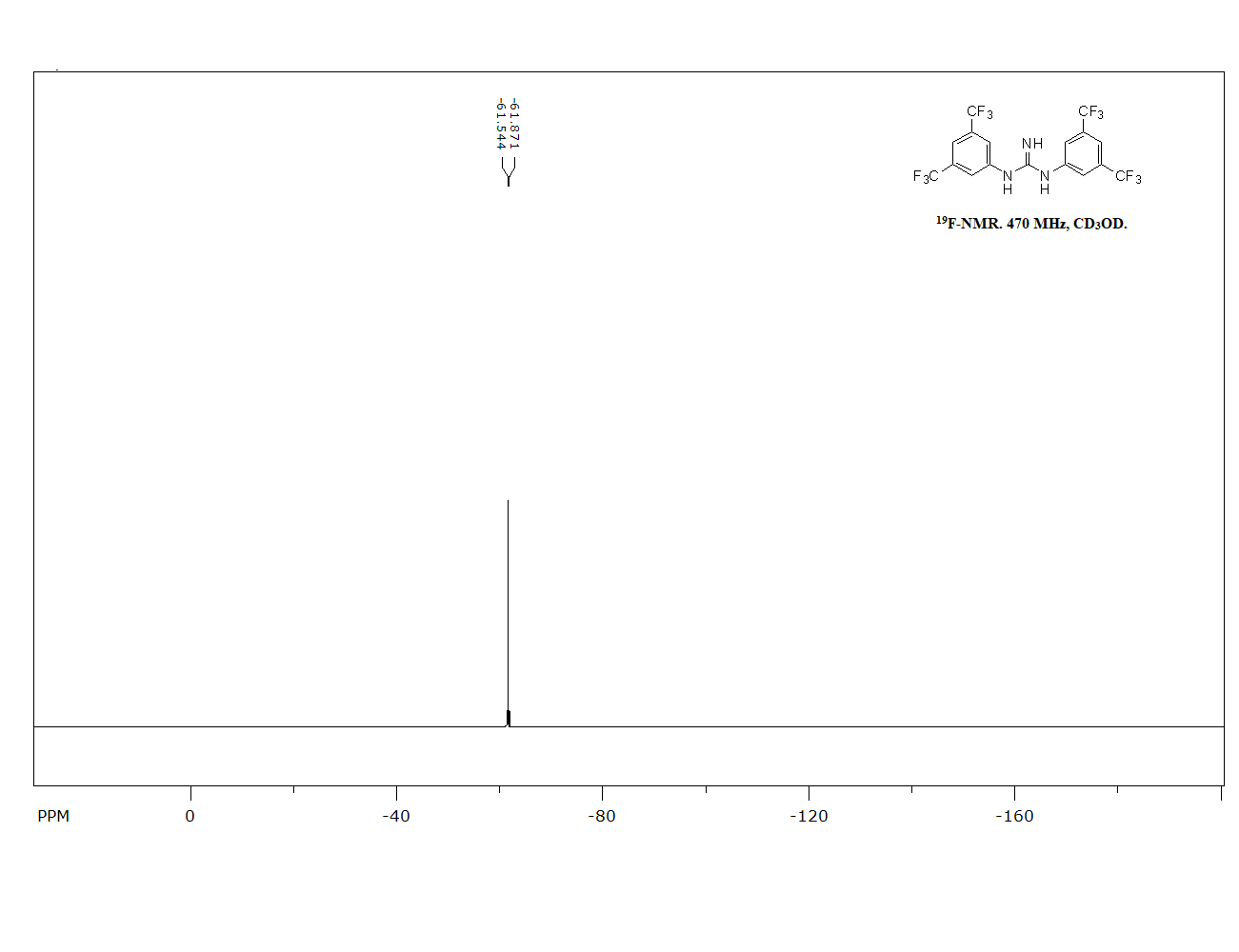


F_IGURE_ 14S: ^19^F-NMR spectrum of the compound 8


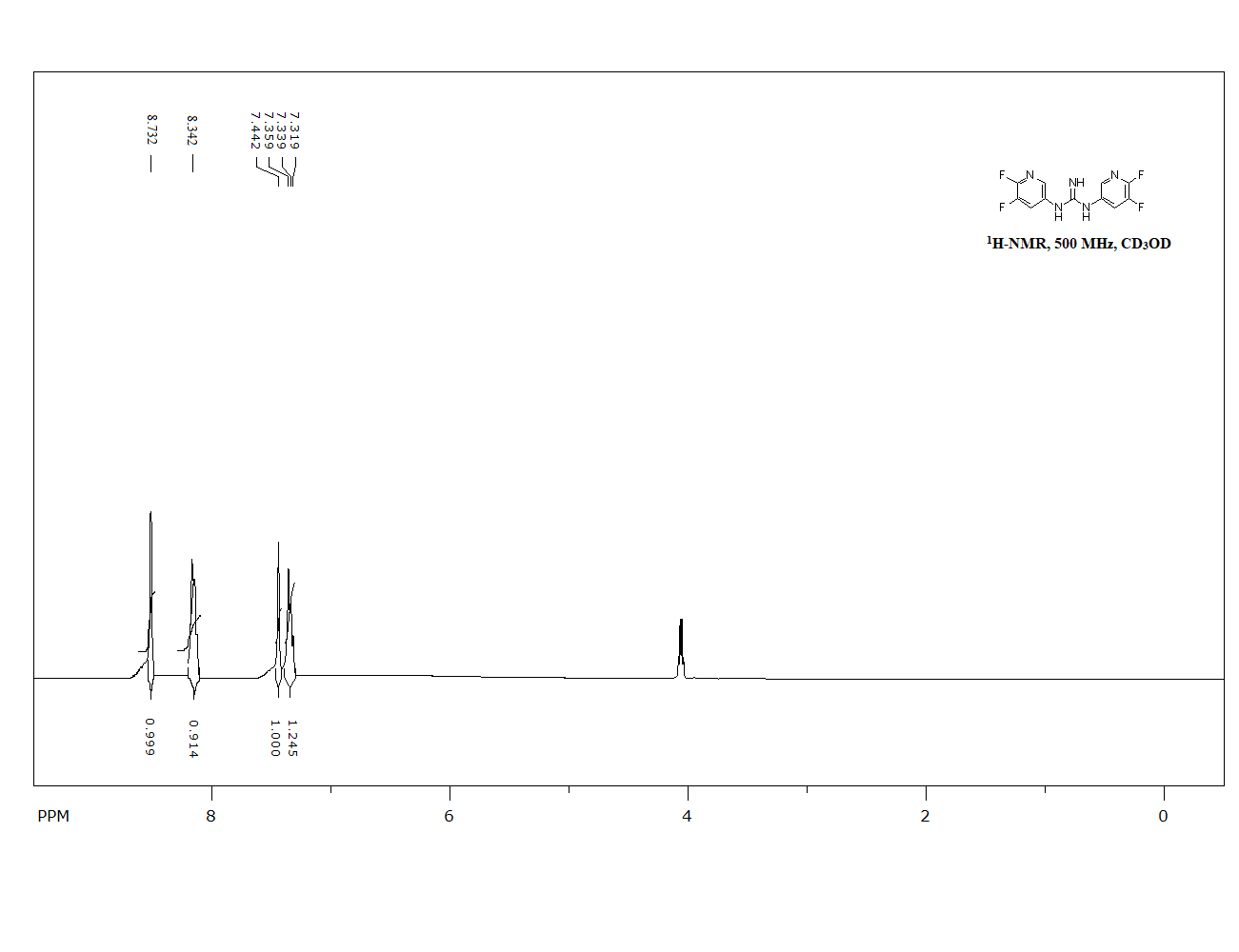


F_IGURE_ 15S: ^1^H-NMR spectrum of the compound 9


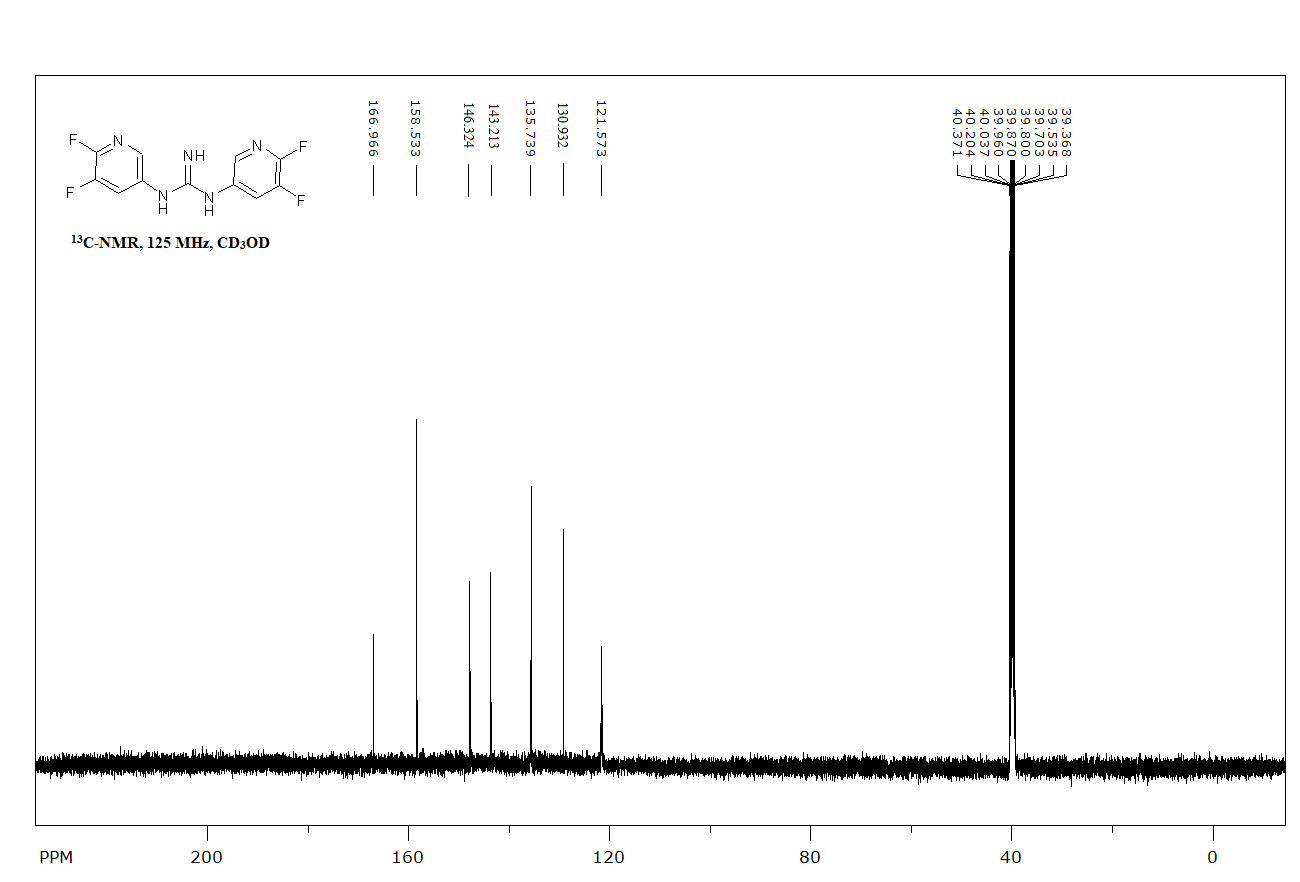


F_IGURE_ 16S: ^13^C-NMR spectrum of the compound 9


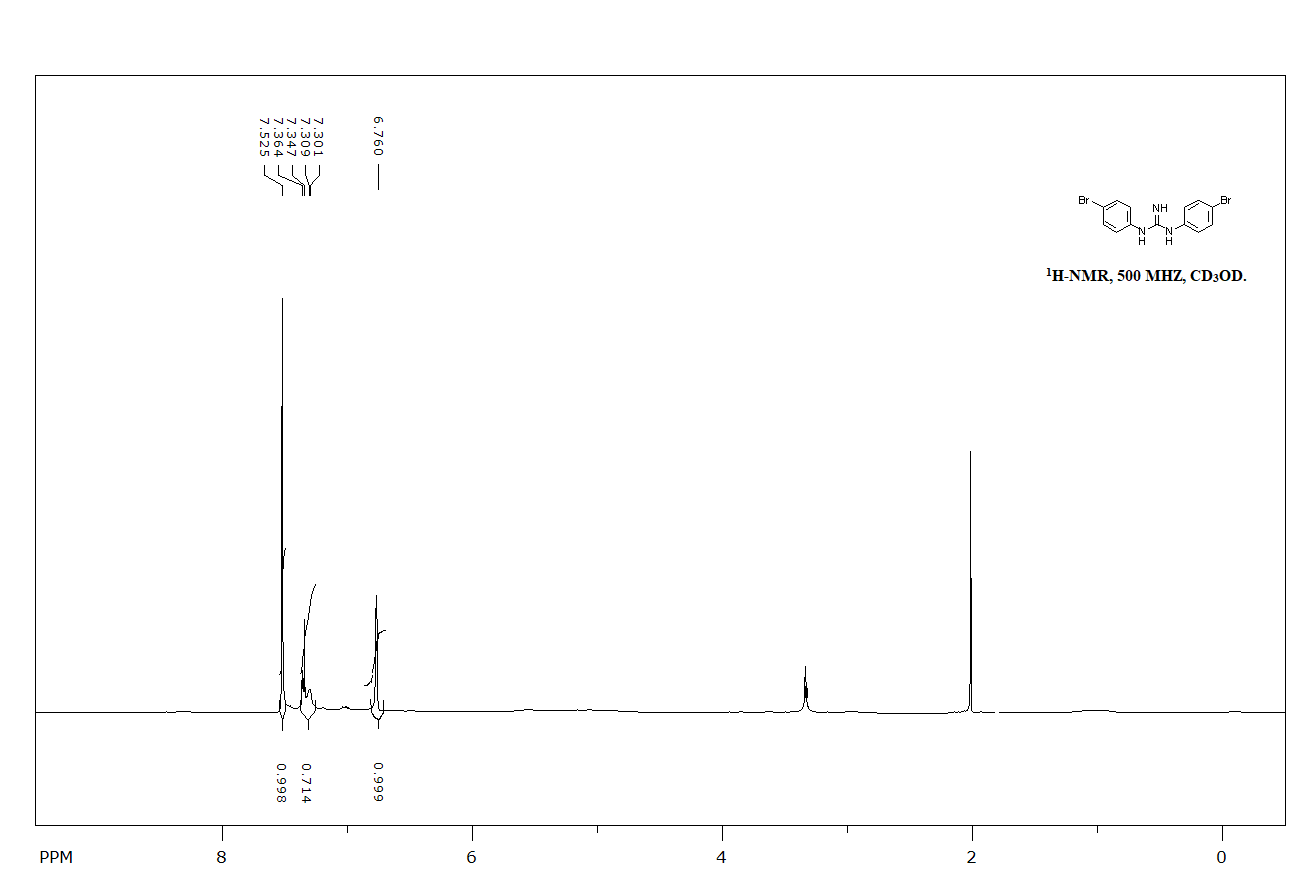


F_IGURE_ 17S: ^1^H-NMR spectrum of the compound 10


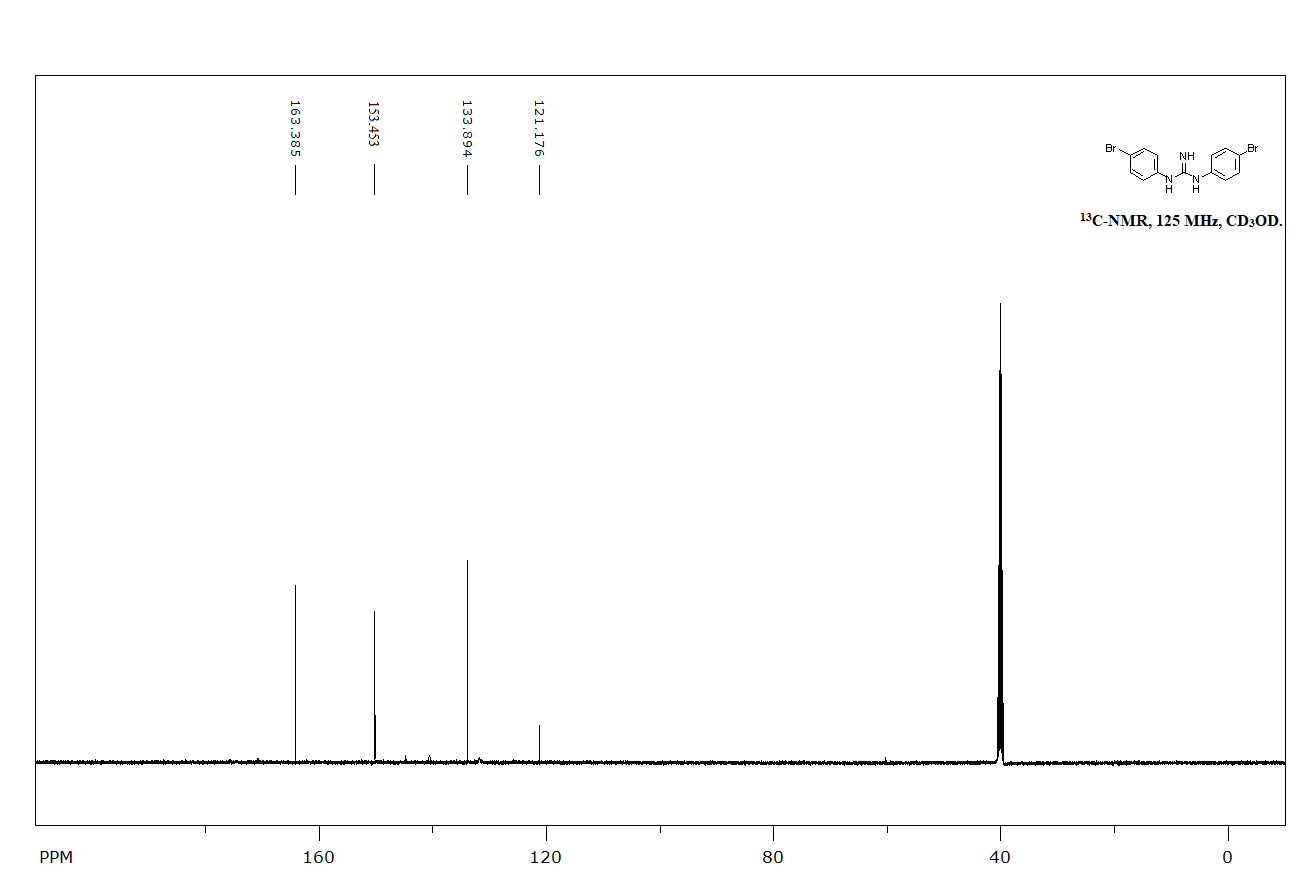


F_IGURE_ 18S: ^13^C-NMR spectrum of the compound 10


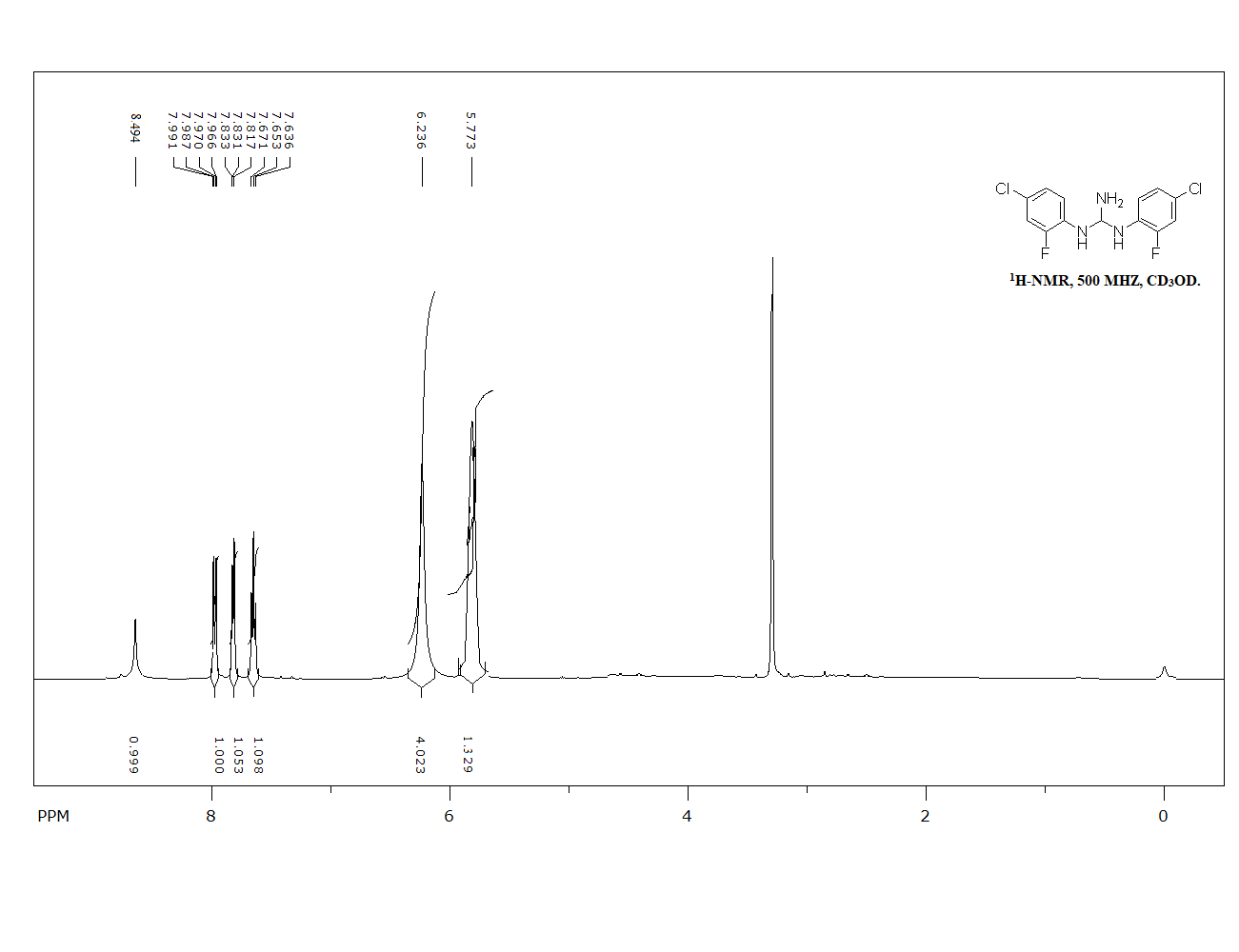


F_IGURE_ 19S: ^1^H-NMR spectrum of the compound 11


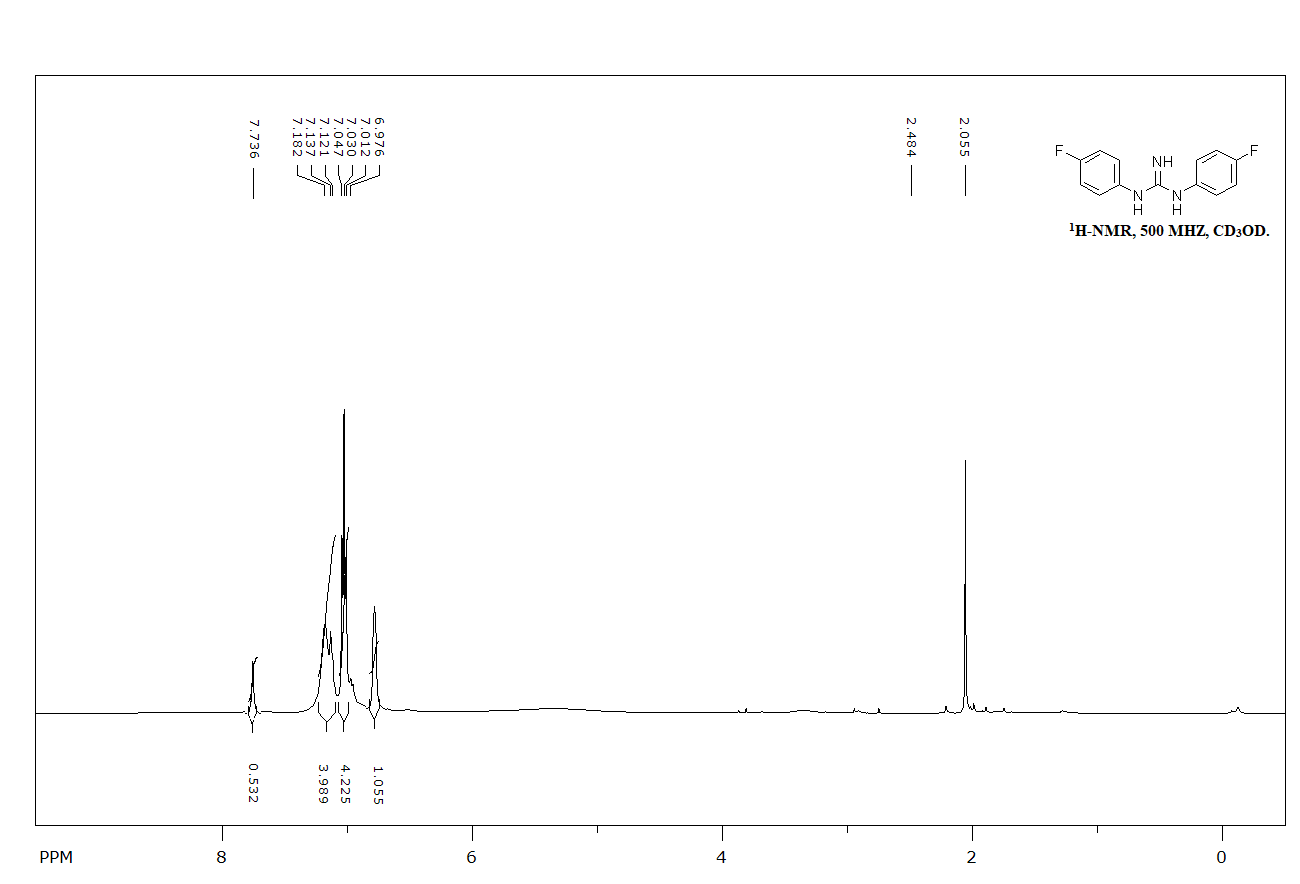


F_IGURE_ 20S: ^1^H-NMR spectrum of the compound 12


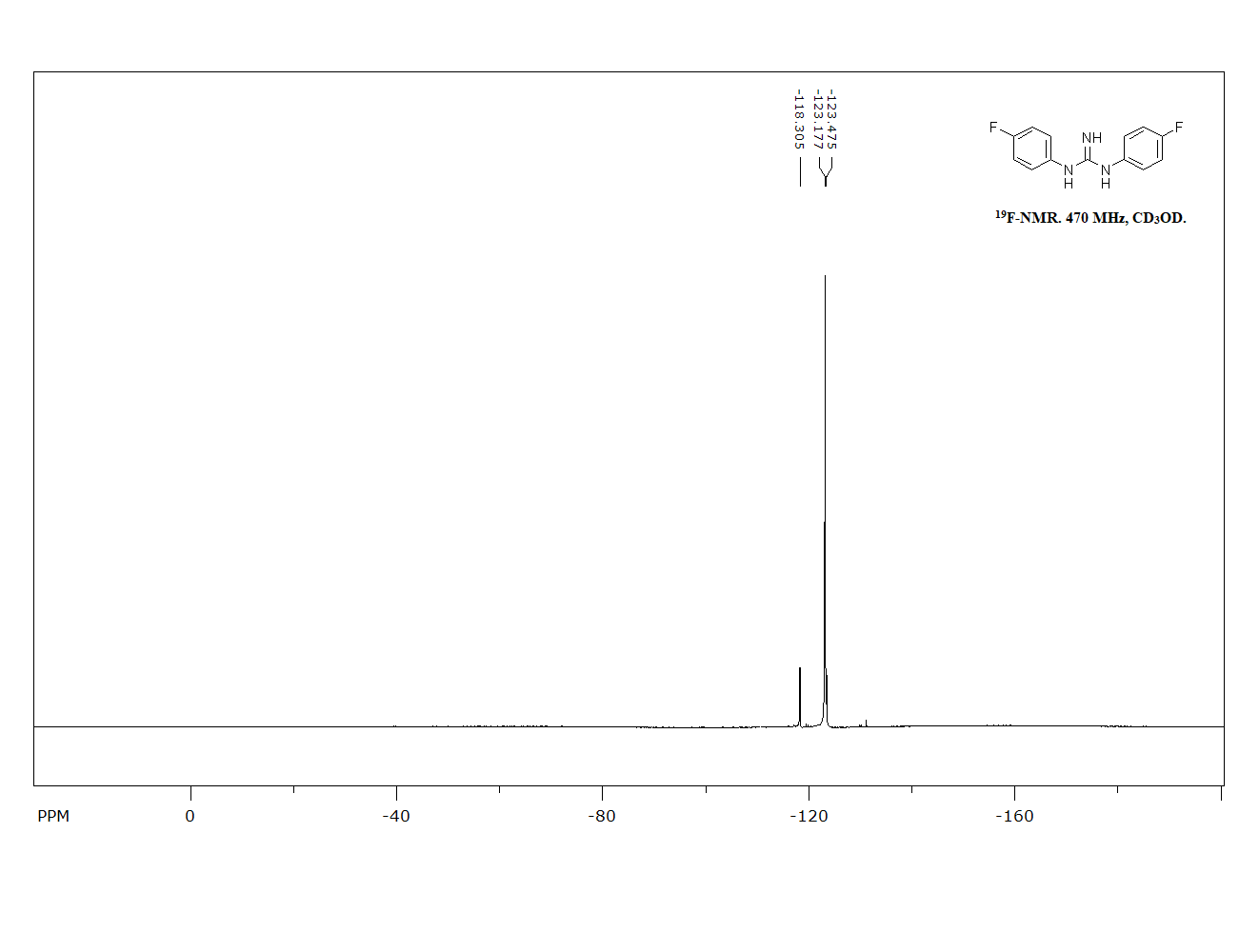


F_IGURE_ 21S: ^19^F-NMR spectrum of the compound 12


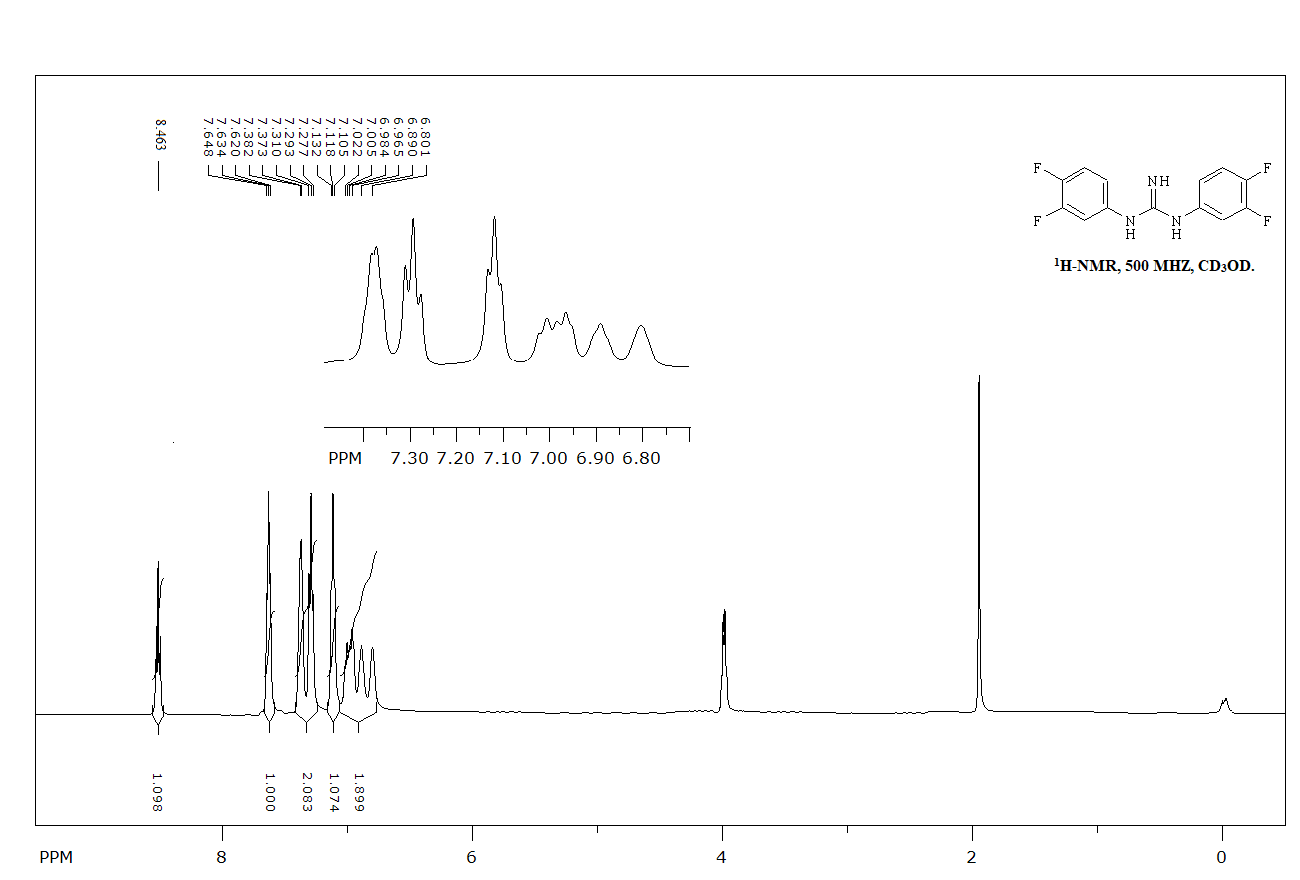


F_IGURE_ 22S: ^1^H-NMR spectrum of the compound 13


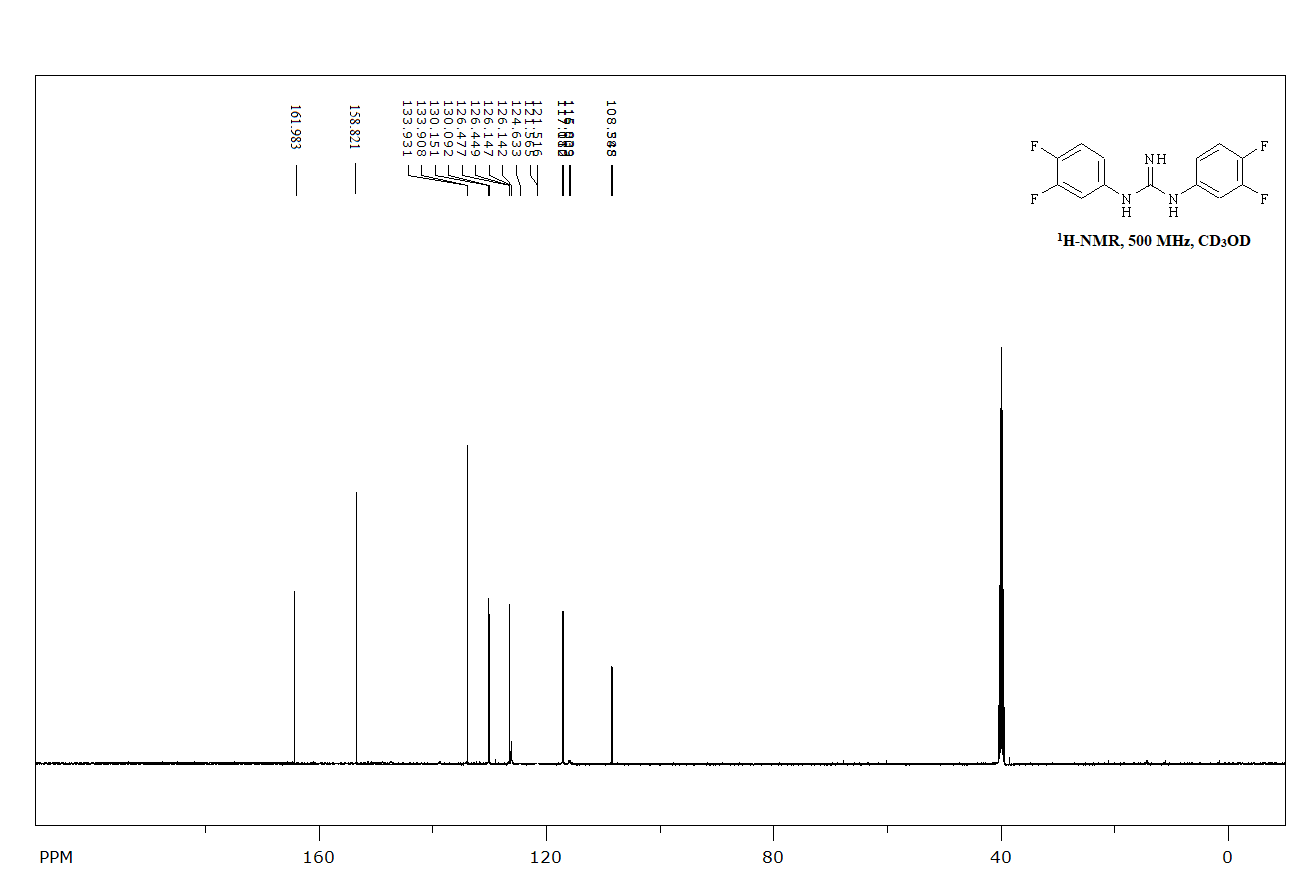


F_IGURE_ 23S: ^13^C-NMR spectrum of the compound 13


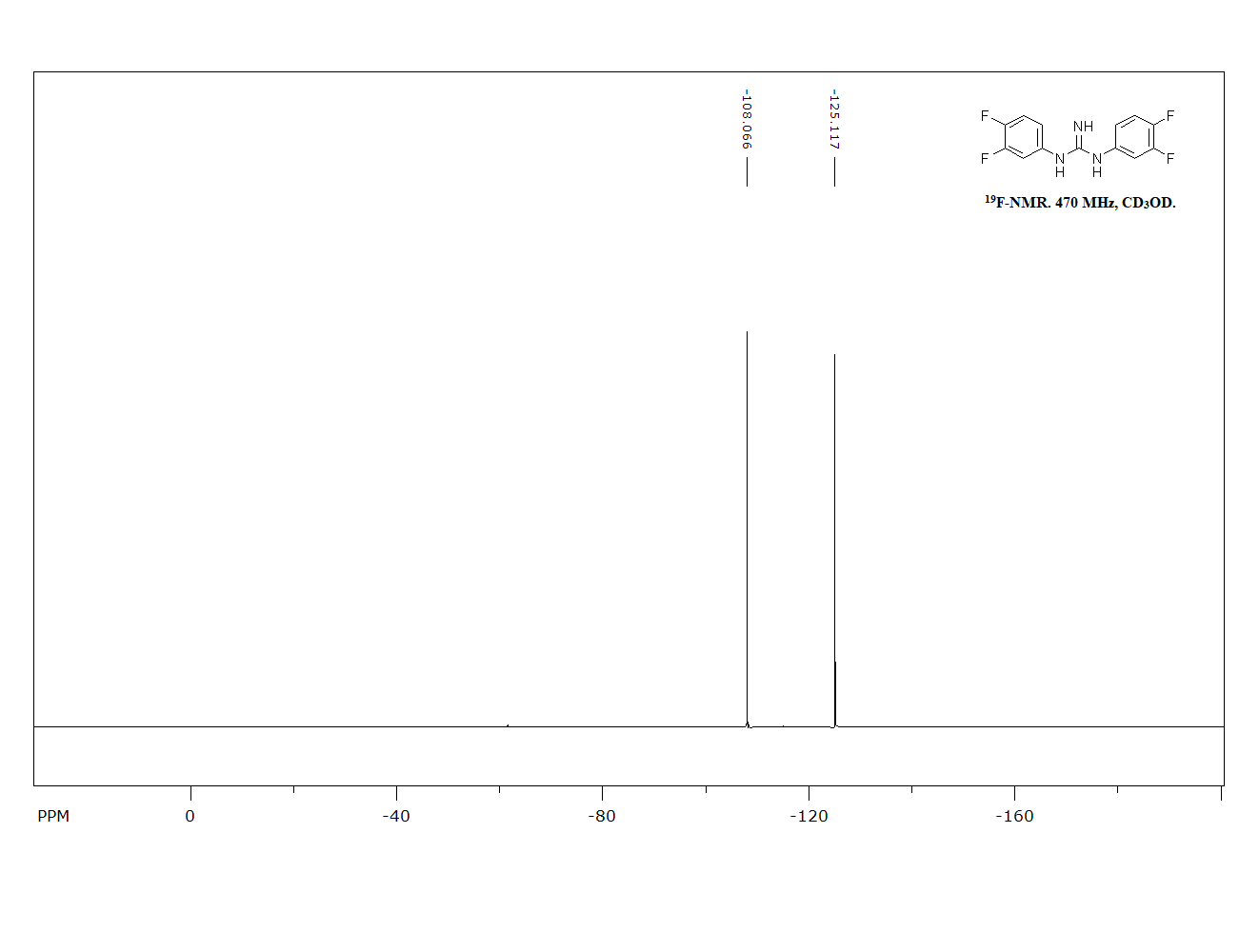


F_IGURE_ 24S: ^19^F-NMR spectrum of the compound 13


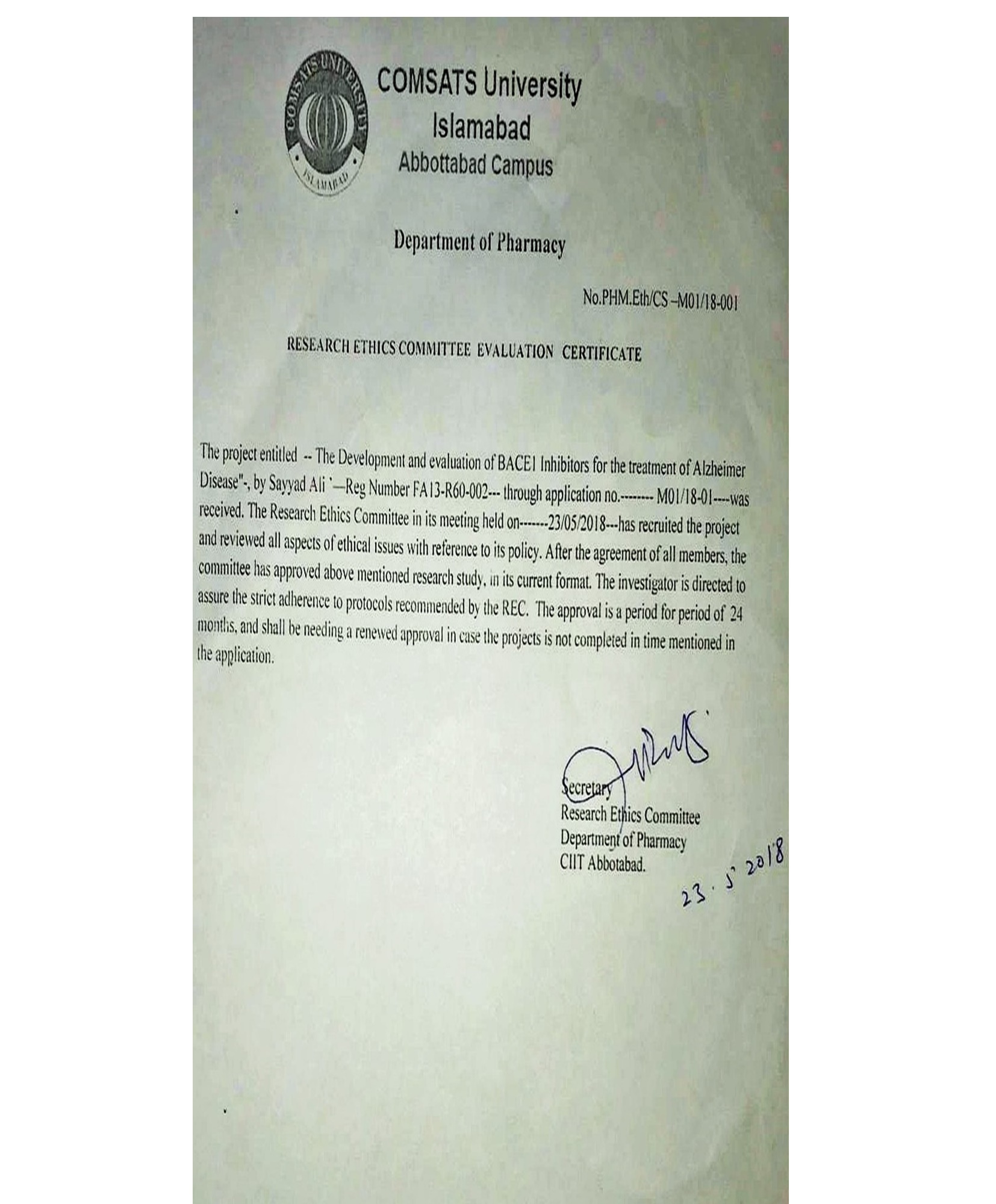

Supplement: Supplementary Materials — Figure 1S: 1H-NMR spectrum of compound (1). Figure 2S: 13C-NMR spectrum of compound (1). Figure 3S: 19F-NMR spectrum of compound (1). Figure 4S: 1H-NMR spectrum of compound (2). Figure 5S: 1H-NMR spectrum of compound (3). Figure 6S: 13C-NMR spectrum of compound (3). Figure 7S: 1H-NMR spectrum of compound (4). Figure 8S: 1H-NMR spectrum of compound (5). Figure 9S: 1H-NMR spectrum of compound (6). Figure 10S: 19F-NMR spectrum of compound (6). Figure 11S: 1H-NMR spectrum of compound (7). Figure 12S: 13C-NMR spectrum of compound (7). Figure 13S: 1H-NMR spectrum of compound (8). Figure 14S: 19F-NMR spectrum of compound (8). Figure 15S: 1H-NMR spectrum of compound (9). Figure 16S: 13C-NMR spectrum of compound (9). Figure 17S: 1H-NMR spectrum of compound (10). Figure 18S: 13C-NMR spectrum of compound (10). Figure 19S: 1H-NMR spectrum of compound (11). Figure 20S: 1H-NMR spectrum of compound (12). Figure 21S: 19F-NMR spectrum of compound (12). Figure 22S: 1H-NMR spectrum of compound (13). Figure 23S: 13C-NMR spectrum of compound (13). Figure 24S: 19F-NMR spectrum of compound (13). [file 8934289.f1.docx]
